# A new data assimilation method for high-dimensional models

**DOI:** 10.1371/journal.pone.0191714

**Published:** 2018-02-08

**Authors:** Guangjie Wang, Xiaoqun Cao, Xun Cai, Jingzhe Sun, Xiaoyong Li, Heng Wang

**Affiliations:** 1 College of Meteorology and Oceanology, National University of Defense Technology, Changsha 410073, China; 2 College of Computer, National University of Defense Technology, Changsha 410073, China; Lanzhou University of Technology, CHINA

## Abstract

In the variational data assimilation (VarDA), the typical way for gradient computation is using the adjoint method. However, the adjoint method has many problems, such as low accuracy, difficult implementation and considerable complexity, for high-dimensional models. To overcome these shortcomings, a new data assimilation method based on dual number automatic differentiation (AD) is proposed. The important advantages of the method lies in that the coding of the tangent-linear/adjoint model is no longer necessary and that the value of the cost function and its corresponding gradient vector can be obtained simultaneously through only one forward computation in dual number space. The numerical simulations for data assimilation are implemented for a typical nonlinear advection equation and a parabolic equation. The results demonstrate that the new method can reconstruct the initial conditions of the high-dimensional nonlinear dynamical system conveniently and accurately. Additionally, the estimated initial values can converge to the true values quickly, even if noise is present in the observations.

## Introduction

The accuracy of numerical weather prediction (NWP) depends on exact initial and boundary conditions and perfect prediction models. In recent years, with the development of numerical models, the quality of the initial conditions has become the bottleneck of the accuracy of NWP [1]. To provide accurate and reasonable initial conditions for NWP, data assimilation is an effective method. Data assimilation derives from the objective analysis of initial values, and it has now become a novel technique that effectively uses considerable unconventional sources of information (such as satellite and radar data) [2]. By combining a mathematical model with irregularly distributed observations through a computer program, a coordinated relationship between the data and the model is constructed according to some algorithms, from which the most likely values on regular grid points can be obtained. Therefore, it has minimal errors in the analytical results and can provide initial conditions for prediction models [3]. In addition, data assimilation can not only effectively improve the accuracy of prediction models but also reduce the uncertainty of the initial conditions; therefore, data assimilation occupies an important position in ocean and atmospheric sciences [4]. Currently, the methods of data assimilation are primarily divided into two categories: the first is sequential data assimilation methods [5], such as Kalman filter (KF), extended Kalman filter, ensemble Kalman filter (EnKF) and particle filter. The second is VarDA methods [6–10], including 3D and 4D VarDA methods (3D/4D-Var). The latter is the extension of the former in the time dimension, and its performance is considerably better. In addition, 4D-Var is the most advanced method for ocean and weather prediction, and it has achieved great success in improving the accuracy of numerical predictions. 4D-Var is essentially a large-scale optimization problem, whose constraint conditions are differential equations. When solving the problems, the value of the gradient vector for the cost function needs be provided for optimization algorithms, which can be achieved by the reverse integration of the adjoint model [11, 12]. Since 4D-Var needs to solve the adjoint model, the amount of computation is particularly large. To reduce the computation cost, an incremental method, which requires the tangent-linear model, is often used. However, the tangent-linear and adjoint models are essentially a first-order differential mode of the nonlinear physical system equation. For complex models, the development of differential models in a manual way is a difficult task [11, 12], and the incremental method does not guarantee the convergence of the results. Moreover, the coding of the adjoint operator is a very complex and heavy task, and the parametrization of the physical process will lead to discontinuity of the cost function.

In fact, the key issue of VarDA to be solved is to calculate the partial derivative of the cost function on control variables (such as the initial value or certain parameters). Generally, there is an implicit nonlinear relationship between the cost function and the unknown parameter, both of which are typically connected by ordinary or partial differential equations. The numerical method that is commonly used to solve partial derivatives is the difference method, but the choice of the difference step is problematic. In particular, for high-dimensional models, the computation cost of the difference method is far greater than that of the adjoint method [12]. Moreover, since the initial values of multiple points are needed, it is easy to generate errors. At present, many data assimilation methods have advantages for low-dimensional nonlinear systems, but for high-dimensional nonlinear systems, their results are not as optimistic. For example, the computation cost is large, the calculation accuracy is not high and even the problems cannot be resolved in some situations. To calculate partial derivatives precisely, Lyness and Moler [13] proposed the complex variable differential method (CVD), whose main idea is to convert the calculation process of derivatives into the calculation process in the field of complex numbers. Cao et al. [14] applied the CVD method to data assimilation problems. A new data assimilation method based on dual number theory was proposed by Cao, Huang et al. [15], and its effectiveness was verified in low-dimensional nonlinear dynamical systems and data assimilation problems of non-differentiable prediction models. However, both aforementioned methods have not been applied to high-dimensional nonlinear dynamical systems and data assimilation problems. Due to the high-dimensional optimization problems whose constraint conditions are nonlinear dynamical prediction models, the gradient of the cost function is difficult to calculate, and thus, a data assimilation method based on dual number AD is proposed. In dual number space, both the function value and the gradient vector value can be accurately obtained by solving differential equations with a numerical solution and calculating the cost function. Therefore, compared to the VarDA method based on the adjoint equation, it has some obvious advantages.

The remainder of this paper is organized as follows. First, the dual number theories and algorithm rules are introduced. Second, a new data assimilation algorithm for high-dimensional numerical models is developed by combining accurate gradient information from dual number AD with the classical optimization algorithm. Third, numerical simulations for data assimilation are implemented for a typical nonlinear advection equation and for a parabolic partial differential equation. Finally, a short conclusion is given.

## Data assimilation based on dual number automatic differentiation

### Introduction of dual numbers

The dual number is defined as:

$$\begin{array}{c} \hat{x}=x+\varepsilon x^{\prime } \end{array}$$ (1)

where *x* is the real part and *x*′ is the dual part. Both *x* and *x*′ are real numbers. The dual label *x*′ does not indicate any specific value but rather the nature of *ε* ≠ 0 and *ε*^*n*^ = 0(*n* > 1) [16, 17]. For convenient expression, Eq (1) can be written in the form such that $\hat{x}=<x,\varepsilon x^{\prime }>$. The equal conditions of two dual numbers are that their real and dual parts are all equal. The dual number is 0 when the real part and the dual part are all 0. The module of a dual number is defined as $|\hat{x}|=x$, which can be positive or negative. The conjugate number of a dual number $\hat{x}$ is ${\hat{x}}^{*}=x-\varepsilon x^{\prime }$; thus, $\hat{x}{\hat{x}}^{*}=x^{2}$. If *x* and *x*′ are extended to a vector, a dual number can be extended to dual vectors. In some special case of data assimilation, the cost function *J*(***x***_0_) is a scalar and ***x***_0_ is a multi-dimensional vector to be estimated; therefore, the dual number is written in the following form: $\hat{x}=x+\varepsilon_{1}x_{1}^{\prime }+\varepsilon_{2}x_{2}^{\prime }+\cdots +\varepsilon_{n}x_{n}^{\prime }=x+\varepsilon \cdot z^{\prime }$. The *ε*_*i*_ of each component in the dual label vector *ε* = (*ε*_1_, *ε*_2_, ⋯, *ε*_*n*_) has the same properties as the dual label *ε*, and *ε*_*i*_*ε*_*j*_ = 0.

The dual number concept and its theory have been proposed for more than 100 years, but until the 1980s, dual number theory was applied to robot kinematics, spatial structures and other kinematic and dynamic problems [18, 19]. In recent years, dual number theory has opened up a new way for calculating the derivative information accurately and efficiently [20, 21].

### Dual number automatic differentiation algorithm

Using the properties of the dual number, the algebraic operations in the real number field can be extended to dual number space. Assume that two dual numbers are $\hat{m}=m+\varepsilon m^{\prime }$ and $\hat{n}=n+\varepsilon n^{\prime }$ and that their sum, difference and multiplication will be as follows [21]:

$$\begin{array}{c} \left(m+m^{\prime }\varepsilon \right)\pm \left(n+n^{\prime }\varepsilon \right)=\left(m\pm n\right)+\left(m^{\prime }\pm n^{\prime }\right)\varepsilon \end{array}$$ (2)

$$\begin{array}{c} \left(m+m^{\prime }\varepsilon \right)\times \left(n+n^{\prime }\varepsilon \right)=mn+mn^{\prime }\varepsilon +nm^{\prime }\varepsilon +m^{\prime }n^{\prime }\varepsilon^{2}=mn+\left(mn^{\prime }+nm^{\prime }\right)\varepsilon \end{array}$$ (3)

In Eq (3), *m*′*n*′*ε*^2^ is omitted, not because *m*′ and *n*′ are too small but because the square of *ε* is 0 [17]. The dual number multiplication represented by Eq (3) is exactly equal, and there are no truncation errors. In the same way, all of the following for dual number algorithms are strictly correct, and there are no approximations or assumptions. Next, considering the dual number polynomial operation, Eq (4) provides a polynomial representation in real number space:

$$\begin{array}{c} P\left(x\right)=p_{0}+p_{1}x+p_{2}x^{2}+\cdots +p_{n}x^{n} \end{array}$$ (4)

where *p*_0_, *p*_1_, *p*_2_, ⋯, *p*_*n*_ are multinomial coefficients. Replacing the real number *x* with the dual number $\hat{x}=<x,x^{\prime }>=x+x^{\prime }\varepsilon$ in Eq (4) and simultaneously using the rules of dual number addition and multiplication, it is easy to prove the formula as follows:

$$\begin{array}{ll} P\left(\hat{x}\right) & =p_{0}+p_{1}\left(x+x^{\prime }\varepsilon \right)+p_{2}{\left(x+x^{\prime }\varepsilon \right)}^{2}+\cdots +p_{n}{\left(x+x^{\prime }\varepsilon \right)}^{n}\\ & =p_{0}+p_{1}x+p_{2}x^{2}+\cdots +p_{n}x^{n}+p_{1}x^{\prime }\varepsilon +2p_{2}xx^{\prime }\varepsilon +\cdots +np_{n}x^{n-1}x^{\prime }\varepsilon \\ & =P\left(x\right)+P^{\left(1\right)}\left(x\right)x^{\prime }\varepsilon \\ & =<P\left(x\right),P^{\left(1\right)}\left(x\right)x^{\prime }> \end{array}$$ (5)

where *P*^(1)^ represents the first-order derivative of the real polynomial *P*(*x*) on a real variable *x*. *x*′ is the seed number, which can take any value. Clearly, if *x*′ = 1, then the dual part is the exact value of the first-order derivative *dP*(*x*)/*dx*. The other basic algebras and new algorithms of standard functions in dual number space are detailed in the literature [21].

For other basic functions, their dual number operation relation can be given by a similar derivation method, and the general two-variable basic function *F* is as follows:

$$\begin{array}{c} F\left(\hat{x},\hat{y}\right)=F\left(<x,x^{\prime }>,<y,y^{\prime }>\right)=<F\left(x,y\right),F_{x}\left(x,y\right)x^{\prime }+F_{y}\left(x,y\right)y^{\prime }> \end{array}$$ (6)

In Eq (6), *F*_*x*_ is the partial derivative of the function *F* on the independent variable *x*. *F*_*y*_ is the partial derivative of the function *F* on the independent variable *y*. Eq (6) can be further extended to the case where the independent variable is the dual number vector $\hat{x}=x+\varepsilon x^{\prime }$:

$$\begin{array}{c} F\left(\hat{x}\right)=F\left(x+\varepsilon x^{\prime }\right)=<F\left(x\right),\nabla F\left(x\right)\cdot x^{\prime }> \end{array}$$ (7)

where ***x*** and ***x***′ ∈ *R^n^* are all multi-dimensional vectors. When the above basic arithmetic operations and functions act on mixed independent variables, such as the dual number < *x*, *x*′ > and real number *c*, the first step is to rewrite the real number *c* into the dual number < *c*, 0 >, and the second is to calculate it according to the above algorithms. The derivative of any function *F*(*x*) at point *x*_0_ can be obtained by calculating *F*(< *x*_0_, 1 >) at < *x*_0_, 1 > in dual number space directly, and the result is < *F*(*x*_0_), *F*′(*x*_0_) >. Likewise, as for the function *F*(*x*), whose independent variable is vector ***x*** ∈ *R^n^*, the directional derivative in direction ***x***′ ∈ *R^n^* at point ***x***_0_ can be derived by calculating function *F*(< ***x***_0_, ***x***′ >) in dual number space directly, and the result is < *F*(***x***_0_), ∇*F*(***x***_0_) · ***x***′ >. In conclusion, the derivative value of independent variables can be obtained simultaneously when calculating the function value in dual number space, which achieves the function of automatic differentiation. At the same time, truncation errors will not be introduced because it avoids the difference operation, which results in machine accuracy when it is implemented on a computer.

### A new data assimilation method

In the derivative computing method based on dual number automatic differentiation, for any cost function *J*(*x*) that takes real variable *x* as the independent variable, a dual variable is first constructed by taking *x* as the real part and taking *εx*′ (the *ε* is the dual label and take *x*′ = 1) as the dual part. Second, the dual variable is substituted into the cost function. Thus, the real variable function *J*(*x*) can be transformed into the dual function $\hat{J}\left(\hat{x}\right)$, which takes the dual number $\hat{x}$ as the independent variable. Finally, the dual function $\hat{J}\left(\hat{x}\right)$ is expanded as a Taylor series:

$$\begin{array}{cc} \hat{J}\left(\hat{x}\right) & =J\left(x\right)+\varepsilon x^{\prime }\frac{J^{\prime }\left(x\right)}{1!}+\varepsilon^{2}{x^{\prime }}^{2}\frac{J^{\prime \prime }\left(x\right)}{2!}+\cdots +{\left(\varepsilon x^{\prime }\right)}^{n}\frac{J^{n}\left(x\right)}{n!}+\cdots \\ & =<J\left(x\right),\nabla_{x}J> \end{array}$$ (8)

Due to *ε*^*n*^ = 0(*n* > 1), high-order remainders that are greater than second order on the right-hand side of Eq (8) can be omitted, and the real part and the dual part on both sides of the equation are strictly equal. It can be shown as follows:

$$\begin{array}{c} J\left(x\right)=\text{Re}\left[\hat{J}\left(\hat{x}\right)\right],\nabla_{x}J=\text{Du}\left[\hat{J}\left(\hat{x}\right)\right] \end{array}$$ (9)

In Eq (9), Re[] and Du[] represent the real part and the dual part of the dual function, respectively. From Eq (9), if we want to calculate the first-order derivative of the cost function on the control variable, we only need to replace the independent variables in the cost function of the real number with the corresponding dual variables. After calculating the value of the function, which is in dual number form, the first-order derivative of the cost function on this independent variable can be obtained by taking its dual part. If there are no other error sources, the accuracy of the first-order derivative for the cost function based on dual number automatic differentiation is not affected by the accuracy of the computer. Therefore, it can be considered that the derivative of the function in Eq (9) is the accurate numerical solution [20, 21].

For the 2-D control vector $x=\left({\hat{x}}_{1},{\hat{x}}_{2}\right)=\left(<x_{1},x_{1}^{\prime }>,<x_{2},x_{2}^{\prime }>\right)$, the dual function $\hat{J}\left(\hat{x}\right)$ is expanded as a Taylor series:

$$\begin{array}{cc} \hat{J}\left(\hat{x}\right)= & J\left(x\right)+\varepsilon {x^{\prime }}_{1}\frac{\nabla_{x_{1}}J\left(x\right)}{1!}+\varepsilon {x^{\prime }}_{2}\frac{\nabla_{x_{2}}J\left(x\right)}{1!}\\ & +\frac{\varepsilon^{2}}{2!}({{x^{\prime }}_{1}}^{2}\frac{\partial^{2}J}{\partial x_{1}^{2}}+{x^{\prime }}_{1}{x^{\prime }}_{2}\frac{\partial^{2}J}{\partial x_{1}\partial x_{2}}+{{x^{\prime }}_{2}}^{2}\frac{\partial^{2}J}{\partial x_{2}^{2}})+\cdots \end{array}$$ (10)

High-order remainders that are greater than second order on the right-hand side of Eq (10) can be omitted by using the property of *ε*^*n*^ = 0(*n* > 1). At the same time, the value of the seed number is $x_{1}^{\prime }=x_{2}^{\prime }=1$; thus, the real part and the dual part on both sides of the equation are strictly equal. It can be shown as follows:

$$\begin{array}{c} J\left(x\right)=\text{Re}\left[\hat{J}\left(\hat{x}\right)\right],\nabla_{x}J=\text{Re}\left[\hat{J}\left(\hat{x}\right)\right] \end{array}$$ (11)

For the multi-dimensional control vector, an expression that is similar to Eq (11) can be obtained.

Dual number differentiation can solve the derivative problem of strongly nonlinear and implicit functions that cannot be solved by the general analytical method, which only requires one forward computation in dual number space. Moreover, for high-dimensional situations, the computation cost and errors are considerably less than in the conventional difference method. After obtaining the cost function gradient of the unknown initial state vector, a suitable descent algorithm is selected to optimize the parameters of each unknown parameter according to the following Eq (12):

$$\begin{array}{c} x_{j}^{i+1}=x_{j}^{i}-\nabla_{x_{j}}J|{}_{x^{i}}\cdot \rho_{j}^{i+1},j=1,2,\cdots ,n \end{array}$$ (12)

**Fig 1:**
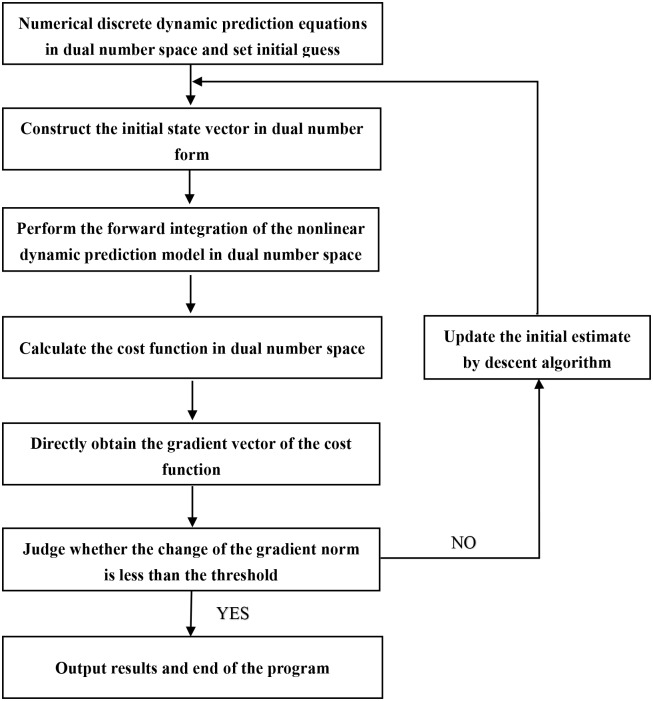
The flow diagram of data assimilation using the dual number AD method.

$\rho_{j}^{i+1}\left(j=1,2,\cdots ,n\right)$ represents the *i*th iteration step, whose size is determined by the descent algorithm. When the value of *i* is 0, ***x*** *^i^* represents the initial guess of the unknown initial state vector. The initial state vector of the high-dimensional nonlinear dynamic prediction model can finally be determined by using Eq (12). The specific process of data assimilation based on dual number automatic differentiation is shown in Fig 1, and the specific processing steps are as follows:

Step 1: First, a difference scheme (such as the upwind scheme) is used to achieve the numerical procedure for solving differential equations. Second, based on dual number rules in dual number space, the relevant procedures (including the prediction model, the observation operator, the cost function and so forth) are modified, which can be suitable for the numerical calculation of dual number space.
Step 2: Provide the initial guess ${\hat{u}}^{0}$ of the initial state vector.
Step 3: Using the provided guess value, the cost function and the gradient vector are calculated by using the automatic differentiation of the dual number theory, and the specific steps are as follows:
  (3a) For each component of the initial state vector, the seed is 1, and the real number is transformed into the dual number. Moreover, the initial state vector is constructed in the form of ${\hat{u}}^{i}=\left(<u_{1}^{i},e_{1}>,<u_{2}^{i},e_{2}>,\cdots ,<u_{n}^{i},e_{n}>\right)$, where ***e****_j_* represents the multi-dimensional unit vector whose *j*th component is 1.
  (3b) The dual number vector ${\hat{u}}^{i}$ is used as the initial condition of the high-dimensional nonlinear dynamic prediction model, and the procedure implemented in the first step is used to perform a forward integration in dual number space. In this way, the evolution track of system state $\hat{x}\left(t\right)$ in dual number form will be obtained, where the real part and the dual part of $\hat{x}\left(t\right)$ represent the value of the state vector ***x***(*t*) and the derivative of ***x***(*t*) on the initial state vector ***u*** at different time steps, respectively.
  (3c) The value of the cost function in dual number space will be calculated by using $\hat{x}\left(t\right)$ and observation data ***y***^*obs*^(*t*). Then, the value of the cost function will be calculated according to Eq (11), whose gradient of the initial state vector component is calculated in the same way.
Step 4: Using the gradient information of the cost function and combined with the typical gradient method, the step size $\rho_{j}^{i+1}$ is obtained. According to Eq (12), the initial state vector of each component is iterated, and the new estimated value ***u***^*i*+1^ is derived. If the termination conditions (such as reaching the convergence accuracy or maximum iteration number that is set in advance) of the procedure are met, the procedure will stop, and the estimated value of the initial state vector will be derived at the same time. If the termination conditions are not met, using the value of the new initial state vector ***u***^*i*+1^, a new iterative loop will start from the third step. To reduce the computation cost, this study uses the usual convergence criterion: the difference of the cost function gradient norm between two consecutive iterations is less than the pre-specified threshold ‖∇_*u*^*i*^_*J*‖ − ‖∇_*u*^*i*+1^_*J*‖ < 10^−6^. In such situations, the calculation error is less than the machine error.

## Results

### Data assimilation for the nonlinear advection equation

To illustrate the usefulness of the new data assimilation method based on dual number automatic differentiation for high-dimensional models, numerical experiments are implemented for the nonlinear advection equation as an example. The nonlinear advection equation [22, 23] is shown as follows:

$$\begin{array}{cc} & \frac{\partial u}{\partial t}+u\frac{\partial u}{\partial x}=0_{}\left(0\leq x\leq L,0\leq t\leq T\right)\\ & u|{}_{x=0}=-3\sin\left(t\pi /6\right) \end{array}$$ (13)

In Eq (13), *u* is a physical quantity, *x* is the horizontal space, and *t* is the time. *L* and *T* are the space and time range of the nonlinear advection equation, respectively. The second equation is the boundary condition. The specific physical significance of this formula can be found in the literature [22, 23]. The standard explicit difference scheme, namely, the upwind scheme, is used to discretize the equations:

$$\begin{array}{cc} & u_{j}^{k+1}=u_{j}^{k}-u\Delta t\left(u_{j}^{k}-u_{j-1}^{k}\right)/\Delta x\\ & u_{0}^{k}=-3\sin\left(k\Delta t\pi /6\right) \end{array}$$ (14)

where the space index of *x* is *j* = 1, 2, ⋯, *J*. *k* = 0, 1, ⋯, *M* is the time index. The variable $u_{j}^{k}$ is used to approximate the physical quantity *u*(*k*Δ*t*, *j*Δ*x*) of discrete points. Moreover, there are Δ*t* = (*T*/*M*) and Δ*x* = (*L*/*J*). The state vector is defined as ***u*** = (*u*_Δ*x*_, *u*_2Δ*x*_, …, *u*_(*N*−1)Δ*x*_, *u*_*N*Δ*x*_)^*T*^. The goal of data assimilation is to estimate the unknown initial state ***u***_0_ = (*u*_0,Δ*x*_, *u*_0,2Δ*x*_, …, *u*_0,(*N*−1)Δ*x*_, *u*_0,*N*Δ*x*_)^*T*^ from a certain number of observation data $u_{i}^{obs}={\left(u_{i,\Delta x}^{obs},u_{i,2\Delta x}^{obs},...,u_{i,(N-1)\Delta x}^{obs},u_{i,N\Delta x}^{obs}\right)}^{T}$ (*i* = 1, 2, ⋯, *N*) for a discrete distribution. In this experiment, the value of *N* is *N* = 12.

**Fig 2:**
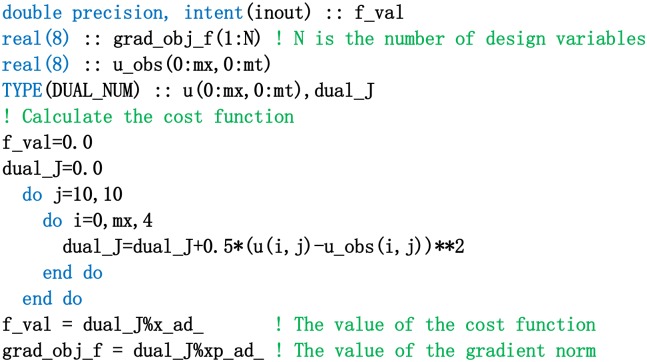
The procedure for calculating the cost function of the nonlinear advection equation in dual number space.

**Fig 3:**
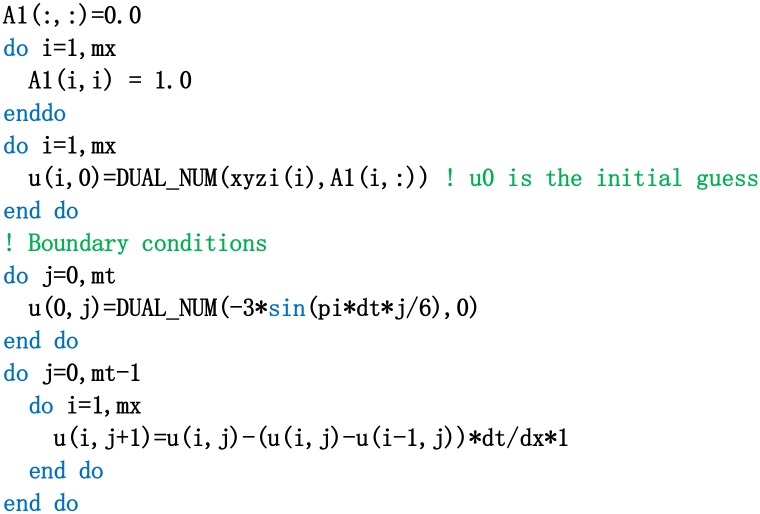
The model procedure of the nonlinear advection equation in dual number space.

In the numerical experiment, the upwind scheme is first used to solve the nonlinear advection equation. Second, the numerical procedure for calculating the cost function (shown in Fig 2) is achieved. Third, its procedure is modified (shown in Fig 3), which is based on dual number rules in dual number space. The acquisition process of the observation data is as follows: the initial state vector is ***u***_0_ = (*u*_0,Δ*x*_, *u*_0,2Δ*x*_, …, *u*_0,(*N*−1)Δ*x*_, *u*_0,*N*Δ*x*_)^*T*^. The true initial value is $u_{0,j\Delta x}^{obs}=3\sin{\left(j\Delta x\pi /6\right)}^{}$ (*j* = 1, 2, …, *N*). The time interval is [0, 0.5], and the time step is Δ*t* = 0.001. The space grid distance is Δ*x* = 1.0. The state value of *u* in discrete time series is derived by numerical integration, and then Gaussian observation noise *N*(0, *σ*_*o*_) are superimposed to form the observation data. The mean value and standard deviation of the noise are 0 and *σ*_0_, respectively. If the background information is not considered, the cost function in discrete form is defined as follows:

$$\begin{array}{c} \hat{J}\left(\hat{u}\right)=\frac{1}{2}\sum_{i=1}^{N}{\left\|u_{i}-u_{i}^{obs}\right\|}^{2} \end{array}$$ (15)

In Eq (15), *N* represents the number of observations, and ‖⋅‖^2^ represents the Euclidean norm. The initial guess of the unknown initial state vector is ***u***_0_ = (1.0, 1.0, ⋯, 1.0). In the process of the iterative estimation for the unknown initial state vector, to update the unknown vector ***u*** every time, the dual number vector ${\hat{u}}_{i}=\left(<u_{i,1}^{},e_{1}>,<u_{i,2}^{},e_{2}>,...,<u_{i,12}^{},e_{12}>\right)$ has to first be constructed, where ***e***_*j*_(*j* = 1, 2, ⋯, 12) represents the 12-D unit vector whose *j*th component is 1, and it is input into the numerical model of the nonlinear advection equation to make a forward (0 → *T*) integration. Second, the cost function $\hat{J}\left(\hat{u}\right)$ is calculated in dual number space. Third, the real part and the dual part are taken as the values of the cost function *J* and the gradient vector ∇_***u***_*J*, respectively. Finally, the conjugate gradient algorithm is used to update the value of the initial state vector [14]. To verify the effectiveness of the new method, the state variables from the 4th time step to the 15th time step are selected as the observation data in the numerical experiment.

**Fig 4:**
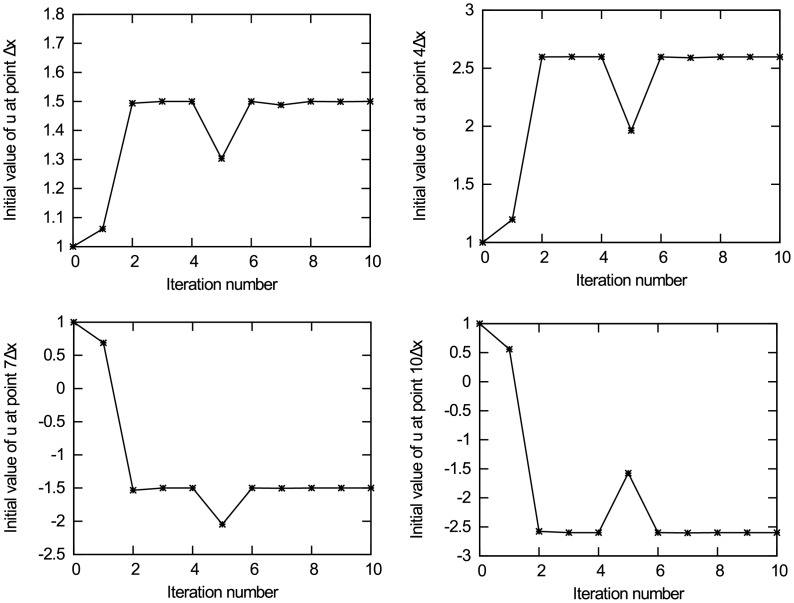
The change of initial state u_0_ with iteration number. (a) Initial state *u*_0,Δ*x*_; (b) Initial state *u*_0,4Δ*x*_; (c) Initial state *u*_0,7Δ*x*_; (d) Initial state *u*_0,10Δ*x*_.

**Fig 5:**
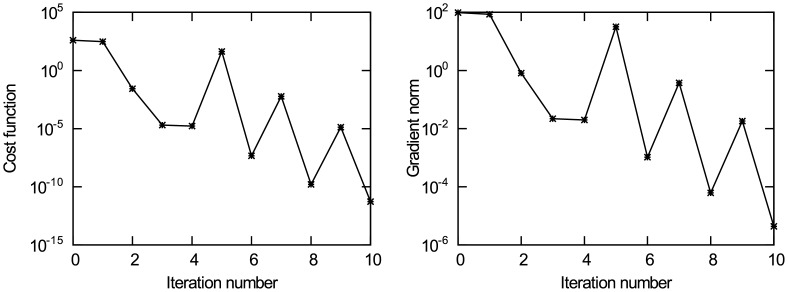
The change of the cost function and gradient norm with iteration number. (a) The cost function *J*(**u**); (b) The gradient norm ‖∇_*u*_*J*‖.

Figs 4 and 5 present the experimental results of data assimilation for the nonlinear advection equation without observation noise. Fig 4a and 4b present the change of the value for unknown initial state quantities *u*_0,Δ*x*_, *u*_0,4Δ*x*_, *u*_0,7Δ*x*_ and *u*_0,10Δ*x*_ in the iterative estimation process, respectively. It can be observed from the figures that the iterative value converges to the true value, and the other eight points have similar results. To avoid redundancy, the results of the other eight points are not presented. Fig 5a shows the change of the cost function *J*(*u*) with iteration number, and Fig 5b shows the change of the gradient norm ‖∇_*u*_*J*‖ with iteration number. It can be observed from the figure that the cost function achieves convergence after the 10th iteration. The value of the unknown initial state vector ***u*** is rounded to the 6th decimal place. The results are presented in Table 1. As shown in Table 1, the estimated value is very close to the true value. Theoretically, when the gradient norm of the cost function on control variables is 0, the unknown initial state vector can take the optimal estimate. By analysing the experimental results, the following conclusions are obtained: in the case without background information and only using minimal observation information, the data assimilation method based on dual number automatic differentiation can accurately estimate the initial state of the nonlinear advection equation, and its estimation accuracy reaches *O*(10^−6^).

**Table 1:** The results of data assimilation for the nonlinear advection equation under different levels of observation noise (The iteration number is 10).

	*u*_0,Δ*x*_	*u*_0,2Δ*x*_	*u*_0,3Δ*x*_	*u*_0,4Δ*x*_	*u*_0,5Δ*x*_	*u*_0,6Δ*x*_	*J*
True value	1.5000000	2.5980763	3.0000000	2.5980762	1.5000002	-0.0000002	∖
*σ*_*o*_ = 0.0	1.5000000	2.5980763	3.0000000	2.5980763	1.5000002	-0.0000004	5.3×10^−12^
*σ*_*o*_ = 0.1	1.5129420	2.5967695	3.0089850	2.5938779	1.4849668	0.0041435	0.6717583
*σ*_*o*_ = 0.2	1.4535858	2.6274088	2.9734110	2.5785737	1.4725619	-0.014866	2.8869303
	*u*_0,7Δ*x*_	*u*_0,8Δ*x*_	*u*_0,9Δ*x*_	*u*_0,10Δ*x*_	*u*_0,11Δ*x*_	*u*_0,12Δ*x*_	*J*
True value	-1.5000000	-2.5980763	-3.0000000	-2.5980763	-1.4999993	-0.0000005	∖
*σ*_*o*_ = 0.0	-1.5000002	-2.5980764	-3.0000000	-2.5980765	-1.4999992	0.0000005	5.3×10^−12^
*σ*_*o*_ = 0.1	-1.4738869	-2.6116443	-2.9905323	-2.6209158	-1.4825408	0.0177094	0.6717583
*σ*_*o*_ = 0.2	-1.5067966	-2.6231179	-3.0218678	-2.5734933	-1.5258349	-0.0271324	2.8869303

Table 1 shows the estimated value of the initial condition, the final value of the cost function and the iteration number for the nonlinear advection equation under different levels of observation noise. As shown in Table 1, when there are no observation errors, the estimation accuracy of the unknown initial states is the highest. Moreover, each initial state quantity can be accurate to the 6th decimal place, which verifies the effectiveness of the data assimilation method based on dual number automatic differentiation in estimating the unknown initial state for high-dimensional nonlinear physical systems. In addition, with increasing observation errors, the accuracy of data assimilation decreases, but the iterative estimation results still converge to the true value. When the standard deviation of the observation errors is *σ*_*o*_ = 0.2, the results of data assimilation are still close to the true value, and it can be accurate to the first decimal place, which demonstrates that the new data assimilation method based on dual number automatic differentiation is capable of removing noise in the observations when working with high-dimensional models.

It is noteworthy that Figs 4 and 5 show a spike in the iteration process. This is because the descent algorithm obtains the iteration step according to the gradient information of the cost function every iteration, the iteration step is not a constant. If the iteration step is not appropriate in the iteration process, there may be a spike. In the next iteration, the descent algorithm will adjust the iteration step according to the gradient information of the cost function until the difference of the cost function gradient norm between two consecutive iterations is less than the pre-specified threshold. The spike is due to the descent algorithm and not due to the dual number approach, and it will not influence the final convergence result.

### Data assimilation for the parabolic equation

To further illustrate the usefulness of the new data assimilation method for high-dimensional models, this part takes the heat conduction equation as an example. This equation [24] is shown as follows:

$$\begin{array}{cc} & \frac{\partial u}{\partial t}=\sigma \frac{\partial^{2}u}{\partial x^{2}},\left(0<x<L,0<t<T\right)\\ & u|{}_{x=0}=0,u|{}_{x=L}=0 \end{array}$$ (16)

In Eq (16), *u* is the state variable of temperature and *σ* is the heat conduction coefficient, which is a constant. The specific physical significance of this formula can be found in the literature [24]. The discrete heat conduction equation is as follows:

$$\begin{array}{l} u_{j}^{k+1}=u_{j}^{k}+\sigma \Delta t\left(u_{j-1}^{k}-2u_{j}^{k}+u_{j+1}^{k}\right)/\Delta x^{2}\\ u_{0}^{k}=0,u_{J}^{k}=0 \end{array}$$ (17)

where the variable $u_{j}^{k}$ is used to approximate the state variable of the temperature *u*(*k*Δ*t*, *j*Δ*x*) of discrete points. The state vector and the goal of data assimilation are the same as the first experiment, but the value of *N* is *N* = 6 in this experiment.

In the numerical experiment, the procedure, the cost function, the acquisition process of the observations and the process of the iterative estimation for the unknown initial state vector are the same as the first experiment. But the true initial value is $u_{0,j\Delta x}^{obs}=\sin{\left(j\Delta x\pi \right)}^{}$ (*j* = 1, 2, …, *N*). The value of the heat conduction coefficient *σ* is 1. The time interval is [0, 0.1], and the time step and the space grid are Δ*t* = 0.001 and Δ*x* = 0.25, respectively. Additionally, the initial guess of the initial state vector is ***u***_0_ = (2.0, 2.0, ⋯, 2.0). To verify the effectiveness, the state variables from the second time step to the 7th time step are selected as the observation data in the numerical experiment.

**Fig 6:**
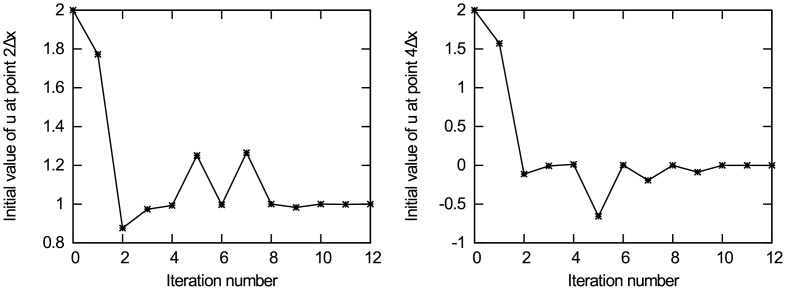
The change of initial state u_0_ with iteration number. (a) Initial state *u*_0,2Δ*x*_; (b) Initial state *u*_0,4Δ*x*_.

**Fig 7:**
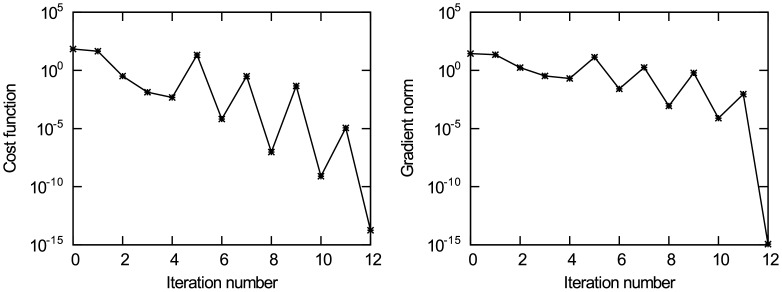
The change of the cost function *J*(u) and gradient norm ‖∇_*u*_*J*‖ with iteration number. (a) The cost function *J*(**u**); (b) The gradient norm ‖∇_*u*_*J*‖.

Figs 6 and 7 present the experimental results of data assimilation for the heat conduction equation without observation noise. Fig 6a and 6b present the change of the value for unknown initial state quantities *u*_0,2Δ*x*_ and *u*_0,4Δ*x*_ in the iterative estimation process, respectively. It can be observed from the figures that the iterative value converges to the true value, and the other four points have similar results. Fig 7a and 7b show the change of the cost function *J*(*u*) and the gradient norm ‖∇_*u*_*J*‖ with iteration number, respectively. It can be seen from the figure that the cost function achieves convergence after the 12th iteration. It is noteworthy that Figs 6 and 7 also show a spike caused by the same reason as the first experiment. The value of the unknown initial state vector ***u*** is rounded to the 7th decimal place. The results are shown in Table 2, from which it can be observed that the estimated value is very close to the true value. By analysing the experimental results, the following conclusions are obtained: in the case of no background information and only using minimal observation information, the new method can accurately estimate the initial state of the heat conduction equation, and its estimation accuracy reaches *O*(10^−7^), which basically can be used as true values. In addition, with increasing observation errors, the accuracy of data assimilation decreases, but the iterative estimation results still converge to the true value. When the standard deviation of observation errors is *σ*_*o*_ = 0.2, the data assimilation results are still close to the true value,which can be accurate to the first decimal place. Therefore, this experiment further verifies the effectiveness and the ability of removing noise in the observations of the new data assimilation method when working with high-dimensional models.

**Table 2:** The results of data assimilation for the heat conduction equation under different levels of observation noise (The iteration number is 12).

	*u*_0,Δ*x*_	*u*_0,2Δ*x*_	*u*_0,3Δ*x*_	*u*_0,4Δ*x*_	*u*_0,5Δ*x*_	*u*_0,6Δ*x*_	*J*
True value	0.7071068	1.0000000	0.7071068	-0.0000001	-0.7071069	-1.0000000	∖
*σ*_*o*_ = 0.0	0.7071068	1.0000000	0.7071068	-0.0000001	-0.7071069	-1.0000000	1.8×10^−14^
*σ*_*o*_ = 0.1	0.7313267	1.0253142	0.6961767	-0.0277506	-0.7024659	-1.0014807	0.1361368
*σ*_*o*_ = 0.2	0.6891197	0.9900859	0.7204978	-0.0339592	-0.6830015	-1.0387555	0.6611682

## Conclusion

To overcome the shortcomings of the gradient computation for high-dimensional prediction models in variational data assimilation when using the adjoint method, a new data assimilation method based on dual number automatic differentiation is proposed. By using the dual number automatic differentiation, the process of gradient analysis is transformed to the computation of the cost function in dual number space, which can obtain the value of the gradient vector simply, efficiently and accurately. The important advantages are that the coding of the adjoint model and the reverse integration are no longer necessary and that the values of the cost function and its corresponding gradient vector can be obtained simultaneously by only one forward computation in dual number space. Compared to the direct difference method, the calculation precision is higher, because the new method is not affected by the truncation errors and the cancellation errors. Compared to the adjoint method, the computation cost is less, because the new method not only does not need to develop the first order differential model, but also does not need to reverse integration of the adjoint equation. The results show that the new method can effectively estimate the initial conditions for high-dimensional numerical prediction models and is capable of removing noise. Therefore, the method proposed in this study is a new data assimilation method with strong adaptability.

However, because different optimization algorithms, such as Quasi-Newton method and conjugate gradient method (used in this paper), may have a slight influence on running time, iteration number and storage space, we will do more experiments to choose the most efficient optimization algorithms according to different cases in the future. Moreover, we expect that using more advanced optimization algoithms in this method can improve its efficiency. In addition, the data assimilation effects and advantages of this new method aiming at more high-dimensional and practical numerical prediction models will be carried out in the subsequent research work.

## Supporting information

S1 File The assimilation result for the nonlinear advection equation. (TXT)

S2 File The result of the cost function for the nonlinear advection equation. (TXT)

S3 File The result of the gradient norm for the nonlinear advection equation. (TXT)

S4 File The assimilation result for the parabolic equation. (TXT)

S5 File The result of the cost function for the parabolic equation. (TXT)

S6 File The result of the gradient norm for the parabolic equation. (TXT)

S7 File All the data used in the manuscript. This file Includes all the result and figures used in the manuscript.
(ZIP)
