# Supplementary material for: A new data assimilation method for high-dimensional models
Source: PLoS One. 2018 Feb 8;13(2):e0191714. doi: 10.1371/journal.pone.0191714 (PMC5805242; doi:10.1371/journal.pone.0191714)
Supplement: S7 File — This file Includes all the result and figures used in the manuscript. (ZIP) [file pone.0191714.s007.zip › minor revision/figures/A new Data Assimilation Method for High-dimensional Models.pdf]

A new Data Assimilation Method for High-dimensional Models

Guangjie Wang[1,2*], **Xiaoqun Cao**[1,2], **Xun Cai**[1,2], **Jingzhe Sun**[1,2],

1 Academy of Ocean Science and Engineering, National University of Defense Technology, Changsha 410073, China,
2 School of Computer Science, National University of Defense Technology, Changsha 410073, China

* wanggj11@lzu.edu.cn(GW)

Abstract

In order to overcome the shortcomings of the gradient computation for high-dimensional models, such as low accuracy, difficult implementation and great complexity, in the variational data assimilation (VarDA) when using the adjoint method, a novel data assimilation method based on dual-number automatic differentiation (AD) is proposed. The important advantages are: the coding of the tangent-linear/adjoint model is not necessary anymore, and the value of cost function and its corresponding gradient vector can be attained simultaneously only by one forward computation in dual-number space. The numerical simulations for data assimilation are implemented for a typical nonlinear advection and a parabolic equation respectively. The results show that the new method can reconstruct the initial conditions of the high-dimensional nonlinear dynamical system conveniently and accurately. And the estimated initial values can be convergent to true ones quickly, even if there are noises in the observations.

Introduction

The accuracy of numerical weather prediction (NWP) depends on the exact initial and boundary conditions and perfect prediction models. In recent years, with the development of models, the quality of the initial conditions has become the bottleneck of the accuracy of NWP [1]. And in order to provide accurate and reasonable initial conditions for NWP, data assimilation is an effective method. Data assimilation derives from the objective analysis of initial value, and now has become a novel technique effectively using the enormous unconventional sources of information [2]. Combining a mathematical model with irregularly distributed observations through a computer program, a coordinated relationship between the data and the model is built according to some algorithms, from which can obtain the most likely values on regular grid-points. Therefore, it has minimum errors of analysis results and can provide initial conditions for prediction models [3]. In addition, data assimilation can not only

improve the accuracy of prediction models effectively, but also reduce the uncertainty of initial conditions, therefore data assimilation occupies an important position in ocean and atmospheric science [4]. Currently, the methods of data assimilation are mainly divided into two categories: one is sequential data assimilation method [5], such as Kalman Filter (KF), Extended Kalman Filter, Ensemble Kalman Filter (EnKF) and Particle Filter. Another is VarDA method [6-10], including 3D and 4D VarDA method (3D/4D-Var). The latter is the extension of the former in the time dimension and the performance is much better. 4D-Var is the most advanced method for ocean and weather forecast, which has achieved great success in improving the accuracy of numerical prediction. 4D-Var is essentially a large-scale optimization problem, whose constraint conditions are differential equations. When solving the problems, the value of the gradient vector for cost function need be provided for optimization algorithms, which can be achieved by the reverse integration of the adjoint model [11, 12]. Since 4D-Var need to solve the adjoint model, the amount of computation is particularly large. In order to reduce the computation cost, incremental method, which needs the tangent-linear model, is often used. However, the tangent-linear and adjoint model is essentially a first-order differential mode of the nonlinear physical system equation. For complex models, the development of differential models in manual way is a difficult task [11-12], and incremental method does not guarantee the convergence of the results. In addition, the coding of the adjoint operator is a very complex and heavy task, and the parametrization of the physical process will lead to the discontinuity of cost function.

Actually the key issue of VarDA to be solved is to calculate the partial derivative of cost function on control variables (such as the initial value or certain parameters). Usually, there is an implicit nonlinear relationship between the cost function and the unknown parameter, and both usually connected by ordinary or partial differential equations. The numerical method which is commonly used to solve partial derivatives is the difference method, but the choice of difference step is a problem. Especially for high-dimensional models, the computation cost of difference method is far greater than the adjoint method [12]. Besides, since the initial value of multiple points is needed, that is easy to generate errors. At present, many data assimilation methods have the advantage for low-dimensional nonlinear systems, but for high-dimensional nonlinear systems, their results are not so optimistic, and even the situations can not be resolved. In order to calculate partial derivatives precisely, Lyness and Moler [13] proposed the complex variable differential method (CVD) whose main idea is to convert the calculation process of derivatives into the calculation process of the complex number field. Cao et al [14] applied the CVD method to data assimilation problems. A new data assimilation method based on dual-number theory was proposed by Cao, Huang et al. [15], and its effectiveness was verified in low-dimensional nonlinear dynamical systems and data assimilation problems of non-differentiable prediction models. However, both methods have not been applied to high-dimensional nonlinear dynamical systems and data assimilation problems. Due to the high-dimensional optimization problems whose constraint conditions are nonlinear dynamical prediction models, the gradient of cost function is difficult to calculate, a data assimilation method based on dual-number AD is proposed. In dual-number space, the function value and the gradient vector value can be accurately attained by solving differential equations with numerical solution and calculating the cost function. Therefore, compared to the VarDA method based on the adjoint equation, it has some obvious advantages.

This paper is organized as follows. Firstly, the dual-number theories and algorithm rules are introduced. Then, the issues of gradient analysis and computation in VarDA is transformed into the process of calculating cost function numerically in dual-number

space, and the gradient vectors can be obtained at the same time in an easy, efficient and accurate way. Secondly, a new data assimilation algorithm for high-dimensional numerical models is developed by combining accurate gradient information from dual-number AD with the classical optimization algorithm. Finally, numerical simulations for data assimilation are implemented for a typical nonlinear advection and a parabolic partial differential equation, respectively. The results suggest that the method can estimate the initial conditions of high-dimensional problems correctly and effectively.

Data assimilation based on dual-number automatic differentiation

1. The introduction of dual-number

The dual-number is defined as:

$$\hat{x} = x + \varepsilon x' \tag{1}$$

where x is the real part and x' is the dual part. Both x and x' are real numbers. Dual label x' not indicate any specific value, but with the nature of $\varepsilon \neq 0$ and $\varepsilon^n = 0 (n > 1)$ [16, 17]. Dual-number concept and its theory have been proposed for more than 100 years, but until 1980s, dual-number was applied to robot kinematics, spatial structures and other kinematic and dynamic problems [18, 19]. In recent years, dual-number theory has opened up a new way for calculating the derivative information accurately and efficiently [20, 21].

In order to express conveniently, formula (1) can be written in the form of binary pair $\hat{x} =< x, \varepsilon x' >$. The equal conditions of two dual-numbers are their real and dual parts are all equal. The dual-number is 0, when the real part and the dual part are all 0. The module of dual-number is defined as $|\hat{x}| = x$, which can be positive or negative. The conjugate number of a dual-number $\hat{x}$ is $\hat{x}^* = x - \varepsilon x'$, so there is $\hat{x}\hat{x}^* = x^2$ [20, 21]. If x and x' are extended to vector, a dual-number can be extended to dual vectors. In special case of data assimilation, the cost function $J(\boldsymbol{x}_0)$ is a scalar and $\boldsymbol{x}_0$ is an multi-dimensional vector to be estimated, so the dual-number is written in the following form: $\hat{\boldsymbol{x}} = x + \varepsilon_1 x'_1 + \varepsilon_2 x'_2 + \cdots + \varepsilon_n x'_n = x + \boldsymbol{\varepsilon} \cdot \mathbf{z}'$. The ε_i of each component in the dual label vector $\boldsymbol{\varepsilon} = (\varepsilon_1, \varepsilon_2, \cdots, \varepsilon_n)$ has the same properties as the dual label ε, and $\varepsilon_i \varepsilon_j = 0$.

2. Dual-number automatic differentiation

Using the properties of dual-number, the algebraic operation in real number field can be extended to dual-number space. Supposing two dual-numbers are $\hat{m} = m + \varepsilon m'$ and $\hat{n} = n + \varepsilon n'$, and their sum, difference and multiplication are [21]:

$$(m + m'\varepsilon) \pm (n + n'\varepsilon) = (m \pm n) + (m' \pm n')\varepsilon \tag{2}$$

$$(m + m'\varepsilon) \times (n + n'\varepsilon) = mn + mn'\varepsilon + nm'\varepsilon + m'n'\varepsilon^2 = mn + (mn' + nm')\varepsilon \tag{3}$$

In formula (3), $m'n'\varepsilon^2$ is omitted, not because m' and n' are too small, but because the square of ε is 0 [17]. The dual-number multiplication represented by formula (3) is exactly equal, there are no truncation errors. In the same way, all of the following for dual-number algorithms are strictly correct and there are no approximations and assumptions. Next considering the polynomial operation of dual-number, formula (4) gives a polynomial representation in real number space:

$$P(x) = p_0 + p_1 x + p_2 x^2 + \cdots + p_n x^n \tag{4}$$

where $p_0, p_1, p_2, \cdots, p_n$ are multinomial coefficients. Replacing the real number x with the dual-number $\hat{x} =< x, x' >= x + x'\varepsilon$ in formula (4) and using the rules of dual-number addition and multiplication at the same time, it is easy to prove the formula as follows:

$$\begin{aligned} P(\hat{x}) =& p_0 + p_1(x + x'\varepsilon) + p_2(x + x'\varepsilon)^2 + \cdots + p_n(x + x'\varepsilon)^n \\ =& p_0 + p_1 x + p_2 x^2 + \cdots + p_n x^n + p_1 x'\varepsilon + 2p_2 x x'\varepsilon + \cdots + n p_n x^{n-1} x'\varepsilon \\ =& P(x) + P^{(1)}(x) x'\varepsilon \\ =& < P(x), P^{(1)}(x) x' > \end{aligned} \tag{5}$$

where $P^{(1)}$ represents the first order derivative of the real polynomial $P(x)$ on a real variable x. x' is the seed number which can take any value. Obviously, if $x' = 1$, then the dual part is the exact value of first order derivative $dP(x)/dx$. By using formula (5), it is easy to extend the operation of dual-number to the calculation of analytic functions and complex functions, and obtain other basic algebras and new algorithms of standard functions in dual-number space, which are listed as follows [21]:

$$\begin{aligned} &\hat{a} \big/ \hat{b} =< a/b, (a'b - ab') \big/ b^2 >, (b \neq 0) \\ &\sin(\hat{y}) =< \sin(y), y' \cos(y) > \\ &\cos(\hat{y}) =< \cos(y), -y' \sin(y) > \\ &\sinh(\hat{y}) =< \sinh(y), y' \cosh(y) > \\ &\cosh(\hat{y}) =< \cosh(y), y' \sinh(y) > \\ &\exp(\hat{y}) =< \exp(y), y' \exp(y) > \\ &|\hat{y}| =< |y|, y' signy >, (y \neq 0) \\ &\hat{y}^k =< y^k, k y^{k-1} y' >, (y \neq 0) \end{aligned}$$

For other basic functions, their dual-number operation relation can be given by a similar derivation method, and the general two-variable basic function F is as follows:

$$F(\hat{x}, \hat{y}) = F(< x, x' >, < y, y' >) =< F(x, y), F_x(x, y) x' + F_y(x, y) y' > \tag{6}$$

In formula (6), F_x is the partial derivative of the function F on the independent variable x. F_y is the partial derivative of the function F on the independent variable y. Formula (6) can be further extended to the case where the independent variable is the dual-number vector $\hat{\boldsymbol{x}} = \boldsymbol{x} + \varepsilon \boldsymbol{x}'$:

$$F(\hat{\boldsymbol{x}}) = F(\boldsymbol{x} + \varepsilon \boldsymbol{x}') =< F(\boldsymbol{x}), \nabla F(\boldsymbol{x}) \cdot \boldsymbol{x}' > \tag{7}$$

where $\boldsymbol{x}$ and $\boldsymbol{x}' \in R^n$ are all multi-dimensional vectors. When the above basic arithmetic operations and functions act on the mixed independent variables, like the dual-number $< x, x' >$ and real number c, what need to do at first is to rewrite the real number c into dual-number $< c, 0 >$, the second is to calculate it according to the above algorithms. The derivative of any function $F(x)$ at point x_0 can be obtained by calculating $F(< x_0, 1 >)$ at $< x_0, 1 >$ in dual-number space directly, and the result is $< F(x_0), F'(x_0) >$. Likewise, as for the function $F(\boldsymbol{x})$, whose independent variable is vector $\boldsymbol{x} \in R^n$, the directional derivative in direction $\boldsymbol{x}' \in R^n$ at point $\boldsymbol{x}_0$ can be derived by calculating function $F(< \boldsymbol{x}_0, \boldsymbol{x}' >)$ in dual-number space directly, and the result is $< F(\boldsymbol{x}_0), \nabla F(\boldsymbol{x}_0) \cdot \boldsymbol{x}' >$. In conclusion, the derivative value of independent variables can be obtained simultaneously when calculating the function value in dual-number space, which achieves the function of automatic differential. At the same time, truncation errors will not be introduced because it avoids the difference operation, which results in the machine accuracy when it is operated in computer.

3. A new data assimilation method

In the derivative computing method based on dual-number automatic differentiation, for any cost function $J(x)$ which takes real variable x as the independent variable, firstly a dual variable is constructed by taking x as the real part and taking $\varepsilon x'$ (the ε is the dual label and take $x' = 1$) as the dual part. Secondly, the dual variable is substituted into the cost function. Thus, the real variable function $J(x)$ can be transformed to the dual function $\hat{J}(\hat{x})$ which takes the dual-number $\hat{x}$ as the independent variable. Finally, the dual function $\hat{J}(\hat{x})$ is expanded as Taylor series:

$$\begin{aligned}\hat{J}(\hat{x}) &= J(x) + \varepsilon x' \frac{J'(x)}{1!} + \varepsilon^2 x'^2 \frac{J''(x)}{2!} + \cdots + (\varepsilon x')^n \frac{J^n(x)}{n!} + \cdots \\ &=< J(x), \nabla_x J > \end{aligned} \tag{8}$$

Due to $\varepsilon^n = 0(n > 1)$, high-order remainders which are more than second-order in right-hand side of formula (8) can be omitted, the real part and the dual part on both sides of equation are strictly equal. It can be shown as follows:

$$J(x) = \mathrm{Re}[\hat{J}(\hat{x})], \nabla_x J = \mathrm{Du}[\hat{J}(\hat{x})] \tag{9}$$

In formula (9), Re[ ] and Du[ ] represent the real part and the dual part of the dual function respectively. From formula (9), if we want to calculate the first order derivative of cost function on the control variable, we only need to replace the independent variables in cost function of real number with the corresponding dual variables. After calculating the value of function which is in the form of dual-number, the first order derivative of cost function on this independent variable can be obtained by taking its dual part. If there is no other error sources, the accuracy of the first order derivative for cost function based on dual-number automatic differentiation is not affected by the accuracy of the computer. So it can be considered that the derivative of the function in formula (9) is the accurate numerical solution [20, 21].

As for the 2-D control vector $\boldsymbol{x} = (\hat{x}_1, \hat{x}_2) = (< x_1, x_1' >, < x_2, x_2' >)$, the dual function $\hat{J}(\hat{\boldsymbol{x}})$ is expanded as Taylor series:

$$\begin{aligned}\hat{J}(\hat{\boldsymbol{x}}) =& J(\boldsymbol{x}) + \varepsilon {x'}_1 \frac{\nabla_{u_1} J(\boldsymbol{x})}{1!} + \varepsilon {x'}_2 \frac{\nabla_{u_2} J(\boldsymbol{x})}{1!} \\ &+ \frac{\varepsilon^2}{2!} \left({x'}_1{}^2 \frac{\partial^2 J}{\partial x_1^2} + {x'}_1 {x'}_2 \frac{\partial^2 J}{\partial x_1 \partial x_2} + {x'}_2{}^2 \frac{\partial^2 J}{\partial x_2^2} \right) + \cdots \end{aligned} \tag{10}$$

High-order remainders which are more than second-order in the right-hand side of formula (10) can be omitted by using the property of ε. At the same time, the value of the seed number is $x_1' = x_2' = 1$, thus the real part and the dual part on both sides of the equation are strictly equal. It can be shown as follows:

$$J(\boldsymbol{x}) = \mathrm{Re}[\hat{J}(\hat{\boldsymbol{x}})], \nabla_{\boldsymbol{x}} J = \mathrm{Re}[\hat{J}(\hat{\boldsymbol{x}})] \tag{11}$$

As for the multi-dimensional control vector, an expression which is similar to the formula (11) can be obtained.

Dual-number differentiation can solve the derivative problem of the strongly nonlinear and implicit function which cannot be solved by the general analytical method, which only need one forward computation in dual-number space. And for high-dimensional situations, the computation cost and errors are much less than the conventional difference method. In short, the new method calculates the value of cost function, meanwhile it can solve the derivative value on the unknown variables simultaneously. Compared to the direct difference method, the AD method based on dual-number theory is not affected by the truncation errors and cancelation errors, so

the calculation precision is higher. Compared to the adjoint method, the new method not only does not need to develop the first order differential model, such as the tangent-linear model and the adjoint model of the original prediction model, but also does not need to have the reverse integration of the adjoint equation, so the calculation cost is less. Thus, the method avoids the specialized derivative calculation, which can easily achieve the goal of solving the gradient for cost function in data assimilation. After obtaining the cost function gradient of the unknown initial state vector, the suitable descent algorithm is selected to optimize the parameters of each unknown parameter according to the following formula [15]:

$$x_j^{i+1} = x_j^i - \nabla_{x_j} J \,|_{x^i} \cdot \rho_j^{i+1}, j = 1, 2, \cdots, n \tag{12}$$

The initial state vector of the high-dimensional nonlinear dynamic prediction model can be determined at last. When the value of i is 0, $\boldsymbol{x}^i$ represents the initial guess of the unknown initial state vector, $\rho_j^{i+1} (j = 1, 2, \cdots, n)$ represents the ith iteration step, whose size is determined by the descent algorithm. The specific process of data assimilation based on dual-number automatic differentiation is shown in Fig 1, and the specific process is as follows:

The first step: Firstly, a difference scheme (such as upwind scheme) is used to achieve the numerical procedure for solving differential equations. Secondly, based on dual-number rules in dual-number space, the relevant procedures (including the prediction model, the observation operator, the cost function etc.) are modified, which can be suitable for the numerical calculation of dual-number space.

The second step: Give the initial guess $\hat{\boldsymbol{u}}^0$ of the initial state vector.

The third step: Using the given guess value, the cost function and the gradient vector are calculated by using the automatic differentiation of the dual-number theory, and the specific steps are shown as follows:

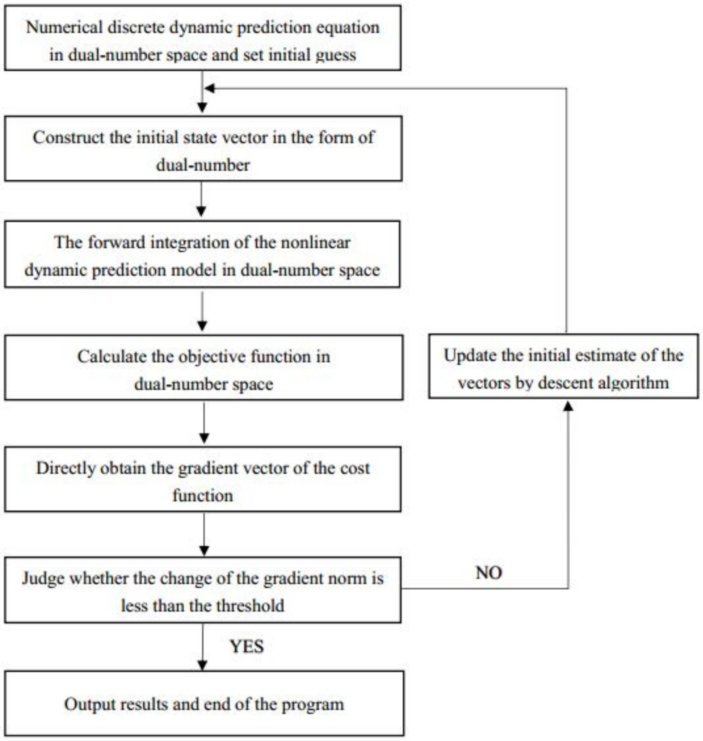

Fig 1. The flow diagram of data assimilation using dual-number AD method.

(1) For each component of the initial state vector, the seed is 1, and the real number is transformed into the dual-number. And the initial state vector is constructed in the form of $\hat{\boldsymbol{u}}^i = (< u_1^i, \boldsymbol{e}_1 >, < u_2^i, \boldsymbol{e}_2 >, \cdots, < u_n^i, \boldsymbol{e}_n >)$, where $\boldsymbol{e}_j$ represents the multi-dimensional unit vector whose jth component is 1.

(2) The dual-number vector $\hat{\boldsymbol{u}}^i$ is used as the initial condition of the nonlinear dynamic prediction model, and the procedure implemented in the first step is used to carry out a forward integration in dual-number space. In this way, the evolution track of system state $\hat{\boldsymbol{x}}(t)$ in the form of dual-number will be obtained, where the real part and the dual part of $\hat{\boldsymbol{x}}(t)$ represent the value of the state vector $\boldsymbol{x}(t)$ and the derivative (or Jacobian matrix) of $\boldsymbol{x}(t)$ on the initial state vector $\boldsymbol{u}$ at different time steps, respectively.

(3) The value of cost function in dual-number space will be calculated by using $\hat{\boldsymbol{x}}(t)$ and observation data $\boldsymbol{y}^{obs}(t)$. Then the value of cost function will be calculated according to formula (11), whose gradient of the initial state vector component takes the same way to calculate.

The fourth step: Using the gradient information of cost function and combined with the typical gradient method, the step size ρ_j^{i+1} is obtained. According to formula (12), the initial state vector of each component is iterated, and the new estimated value $\boldsymbol{u}^{i+1}$ is derived. If termination conditions (such as reaching the convergence accuracy or maximum iteration number which is set in advance) of the procedure are met, the procedure will stop, and the estimation value of the initial state vector will be derived at the same time. If termination conditions are not met, using the value of the new initial state vector $\boldsymbol{u}^{i+1}$, a new iterative loop will start from the third step.

Results

1. The data assimilation for nonlinear advection equation

In order to illustrate the usefulness of the new data assimilation method based on dual-number automatic differentiation to high-dimensional models, numerical experiments are implemented for the nonlinear advection equation as an example. The nonlinear advection equation [22,23] is shown as follows:

$$\begin{aligned} \frac{\partial u}{\partial t} + u\frac{\partial u}{\partial x} &= 0 (0 \le x \le L, 0 \le t \le T) \\ u\,|_{x=0} &= -3\sin(t\pi/6) \end{aligned} \tag{13}$$

In formula (13), u is a physical quantity; x is the horizontal space; t is the time. L and T are the space and time range of the nonlinear advection equation respectively. The second equation is the boundary condition. The specific physical significance of this formula can be found in references. The standard explicit difference scheme, upwind scheme, is used to discrete the equations:

$$\begin{aligned} u_j^{k+1} &= u_j^k - u\Delta t(u_j^k - u_{j-1}^k)/\Delta x \\ u_0^k &= -3\sin(k\Delta t\pi/6) \end{aligned} \tag{14}$$

where the space index of x is $j = 1, 2, \cdots, J$. $k = 0, 1, \cdots, M$ is the time index. The variable u_j^k is used to approximate the physical quantity $u(k\Delta t, j\Delta x)$ of discrete points. And there are $\Delta t = (T/M)$ and $\Delta x = (L/J)$. The state vector is defined as $\boldsymbol{u} = (u_{\Delta x}, u_{2\Delta x}, ..., u_{(N-1)\Delta x}, u_{N\Delta x})^T$. The goal of data assimilation is to estimate the unknown initial state $\boldsymbol{u}_0 = (u_{0,\Delta x}, u_{0,2\Delta x}, ..., u_{0,(N-1)\Delta x}, u_{0,N\Delta x})^T$ from a certain number of observation data $\boldsymbol{u}_i^{obs} = (u_{i,\Delta x}^{obs}, u_{i,2\Delta x}^{obs}, ..., u_{i,(N-1)\Delta x}^{obs}, u_{i,N\Delta x}^{obs})^T$ $(i = 1, 2, \cdots, N)$ for discrete distribution. In this experiment, the value of N is $N = 12$.

In the numerical experiment, firstly, the upwind scheme is used to solve the nonlinear advection equation. Secondly, the numerical procedure for calculating the cost function (shown in Fig 2) is achieved. Thirdly, its procedure is modified (shown in Fig 3), which is based on dual-number rules in dual-number space. The acquisition process of the observation data is as follows: the initial state vector is $\boldsymbol{u}_0 = (u_{0,\Delta x}, u_{0,2\Delta x}, ..., u_{0,(N-1)\Delta x}, u_{0,N\Delta x})^T$. The truth initial value is $u^{obs}_{0,j\Delta x} = 3\sin(j\Delta x\pi/6)$ $(j = 1, 2, ..., N)$. The time interval is [0, 0.5], and the time step is $\Delta t = 0.001$. The space grid distance is $\Delta x = 1.0$. The state value of u in discrete time series is derived by the numerical integration and then Gaussian observation noises $N(0, \sigma_o)$ is superimposed to form the observation data. The mean value and standard deviation of noises are 0 and σ_0 respectively. If the background information is not considered, the cost function of discrete form is defined as follows:

$$\hat{J}(\hat{\boldsymbol{u}}) = \frac{1}{2}\sum_{i=1}^{N} \left\| \boldsymbol{u}_i - \boldsymbol{u}_i^{obs} \right\|^2 \tag{15}$$

In formula (15), N represents the number of observations, and $\|\cdot\|^2$ represents the Euclidean norm. The initial guess of the unknown initial state vector is $\boldsymbol{u}_0 = (1.0, 1.0, \cdots, 1.0)$. In the process of the iterative estimation for the unknown initial state vector, in order to update the unknown vector $\boldsymbol{u}$ every time, firstly, the

```
  double precision, intent(inout) :: f_val
  real(8) :: grad_obj_f(1:N) ! N is the number of design variables
  real(8) :: u_obs(0:mx,0:mt)
  TYPE(DUAL_NUM) :: u(0:mx,0:mt),dual_J
! calculate cost function
  f_val=0.0
  dual_J=0.0
    do j=4,15
      do i=1,mx
        dual_J=dual_J+0.5*(u(i,j)-u_obs(i,j))**2
      end do
    end do
  f_val = dual_J%x_ad_       ! the value of cost function
  grad_obj_f = dual_J%xp_ad_ ! the value of gradient norm
```

Fig 2. The procedure for calculating cost function in dual-number space.

```
 A1(:,:)=0.0
  do i=1,mx
    A1(i,i) = 1.0
  enddo
!
  do i=1,mx
    u(i,0)=DUAL_NUM(u0(i),A1(i,:)) !u0 is the initial guess
  end do
!boundary conditions
  do j=0,mt
    u(0,j)=DUAL_NUM(-3*sin(pi*dt*j/6),0)
  end do
!
  do j=0,mt-1
    do i=1,mx
      u(i,j+1)=u(i,j)-u(i,j)*(u(i,j)-u(i-1,j))*dt/dx
    end do
  end do
```

Fig 3. The model procedure in dual-number space.

dual-number vector $\hat{\boldsymbol{u}}_i = (< u_{i,1}, e_1 >, < u_{i,2}, e_2 >, ..., < u_{i,12}, e_{12} >)$ has to be constructed, $\boldsymbol{e}_j (j = 1, 2, \cdots, 12)$ represents the 12-D unit vector whose jth component is 1, and it is input into the numerical model of the nonlinear advection equation to make a forward $(0 \to T)$ integration. Secondly, the cost function $\hat{J}(\hat{\boldsymbol{u}})$ is calculated in dual-number space. Thirdly, the real part and the dual part are taken as the value of the cost function J and the gradient vector $\nabla_{\boldsymbol{u}} J$, respectively. Finally, the conjugate gradient algorithm is used to update the value of the initial state vector [14]. In order to verify the effectiveness of the new method, the state variables from the 4th time step to the 15th time step are selected as the observation data in the numerical experiment.

Figs 4-9 show the experiment results of data assimilation for nonlinear advection equation without observation noises. Figs 4-7 represent the change of the value for unknown initial state quantities $u_{0,\Delta x}$, $u_{0,4\Delta x}$, $u_{0,7\Delta x}$ and $u_{0,10\Delta x}$ in the iterative estimation process, respectively. It can be seen from the figures that the iterative value converge to the true value, and the other eight points have similar results. In order to avoid redundancy, the results of other eight points are not given. Fig 8 shows the change of cost function $J(u)$ with iteration number, and Fig 9 shows the change of gradient normal $\|\nabla_u J\|$ with iteration number. It can be seen from the figure that the cost function achieves convergence after the 10th iteration. The value of the unknown initial state vector $\boldsymbol{u}$ is rounded to the 6th decimal. The results are shown in Table 1. It can be seen from Table 1 that the estimated value is very close to the true value. Theoretically, when the gradient norm of cost function on control variables is 0, the

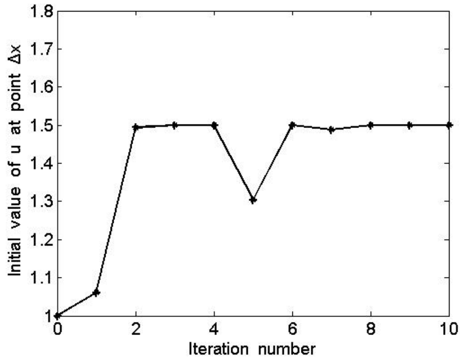

Fig 4. The change of initial state $u_{0,\Delta x}$ with iteration number.

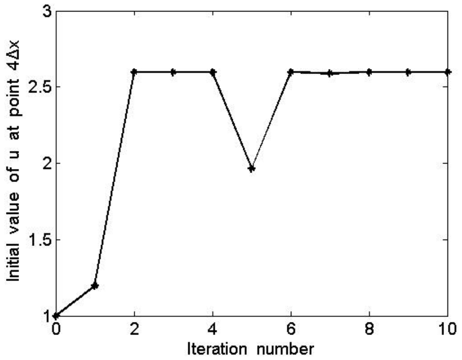

Fig 5. The change of initial state $u_{0,4\Delta x}$ with iteration number.

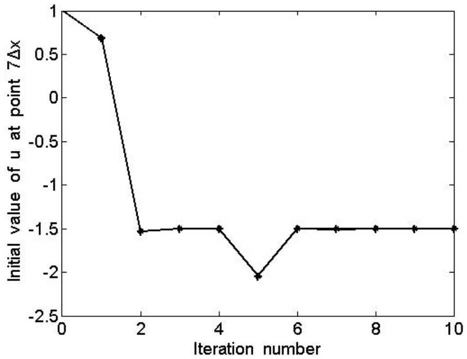

Fig 6. The change of initial state $u_{0,7\Delta x}$ with iteration number.

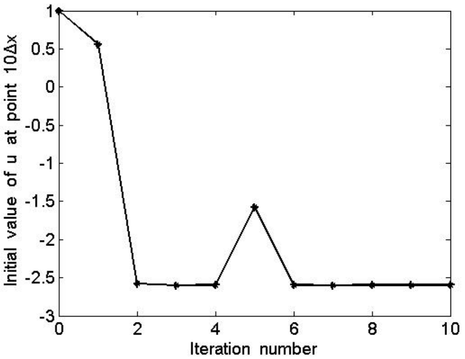

Fig 7. The change of initial state $u_{0,10\Delta x}$ with iteration number.

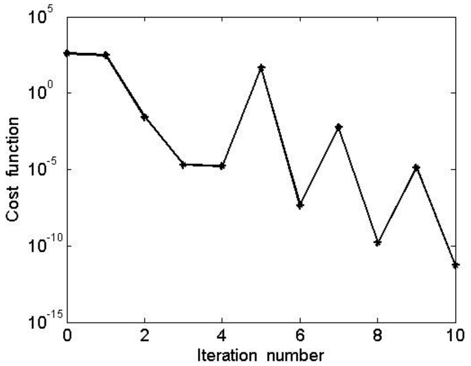

Fig 8. The change of cost function $J(\boldsymbol{u})$ for nonlinear advection equation.

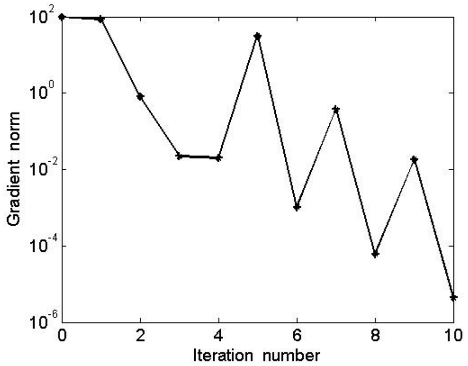

Fig 9. The change of gradient norm $\|\nabla_{\boldsymbol{u}} J\|$ for nonlinear advection equation.

unknown initial state vector can take the optimal estimate. In order to reduce the cost of computation, this study uses the usual convergence criterion: the difference of the cost function gradient norm between two consecutive iterations is less than the pre-specified threshold $\|\nabla_{u^i} J\| - \|\nabla_{u^{i+1}} J\| < 10^{-6}$. At such situations, the calculation error is less than the machine error. By analyzing the experiment results, the conclusions are summarized as follows: in case of without background information and only using little observation information, the data assimilation method based on dual-number automatic differentiation can accurately estimate the initial state of nonlinear advection equation, and its estimation accuracy reaches $O(10^{-6})$.

The advantages of the new assimilation method are that the coding of the adjoint and tangent-linear model are not necessary anymore, and the gradient of cost function can be calculated very conveniently and accurately by using the dual-number automatic differentiation, whose value of cost function can decline rapidly. Table 1 shows the estimated value of initial condition, the final value of cost function and the iteration number for nonlinear advection equation under different levels of observation noises. As Table 1 shows, when there is no observation errors, the estimation accuracy of the unknown initial states is the highest. And each initial state quantity can be accurate to the 6th decimal, which verifies the effectiveness of data assimilation method based on dual-number automatic differentiation in estimating the unknown initial state for high-dimensional nonlinear physical systems. In addition, with the increasing of the observation errors, the accuracy of data assimilation declines, but the iterative estimation results still converge to the true value. When the standard

Table 1. The results of data assimilation for nonlinear advection equation under different levels of observation noises.

	$u_{0,\Delta x}$	$u_{0,\Delta 2x}$	$u_{0,\Delta 3x}$	$u_{0,\Delta 4x}$	$u_{0,\Delta 5x}$	$u_{0,\Delta 6x}$	J	number
True value	1.5000000	2.5980763	3.0000000	2.5980762	1.5000002	-0.0000002	\	\
$\sigma_o = 0.0$	1.5000000	2.5980763	3.0000000	2.5980763	1.5000002	-0.0000004	5.313126E-12	10
$\sigma_o = 0.1$	1.5129420	2.5967695	3.0089850	2.5938779	1.4849668	0.0041435	0.6717583119	10
$\sigma_o = 0.2$	1.4535858	2.6274088	2.9734110	2.5785737	1.4725619	-0.014866	2.886930397	10
	$u_{0,\Delta 7x}$	$u_{0,\Delta 8x}$	$u_{0,\Delta 9x}$	$u_{0,\Delta 10x}$	$u_{0,\Delta 11x}$	$u_{0,\Delta 12x}$	J	number
True value	-1.5000000	-2.5980763	-3.0000000	-2.5980763	-1.4999993	-0.0000005	\	\
$\sigma_o = 0.0$	-1.5000002	-2.5980764	-3.0000000	-2.5980765	-1.4999992	0.0000005	5.313126E-12	10
$\sigma_o = 0.1$	-1.4738869	-2.6116443	-2.9905323	-2.6209158	-1.4825408	0.0177094	0.6717583119	10
$\sigma_o = 0.2$	-1.5067966	-2.6231179	-3.0218678	-2.5734933	-1.5258349	-0.0271324	2.886930397	10

deviation of observation errors is $\sigma_o = 0.2$, the results of data assimilation are still close to the true value, and it can be accurate to the first decimal, which illustrates that the new data assimilation method based on dual-number automatic differentiation is capable of removing noises in the observations when dealing with high-dimensional models.

2. The data assimilation for parabolic equation

In order to further illustrate the usefulness of the new data assimilation method to high-dimensional models, this part takes the heat conduction equation as an example. The equation [24] is shown as follows:

$$\begin{aligned} &\frac{\partial u}{\partial t} = \sigma \frac{\partial^2 u}{\partial x^2}, (0 < x < L, 0 < t < T) \\ &u\,|_{x=0} = 0, \quad u\,|_{x=L} = 0 \end{aligned} \tag{16}$$

In formula (16), u is the state variable of temperature, σ is heat conduction coefficient which is a constant. L and T represent the space and time range for heat conduction equation respectively. The boundary conditions are given in the second equation. The specific physical significance of this formula can be found in references. The discrete heat conduction equation is as follows:

$$\begin{aligned} &u_j^{k+1} = u_j^k + \sigma \Delta t (u_{j-1}^k - 2u_j^k + u_{j+1}^k)/\Delta x^2 \\ &u_0^k = 0, u_J^k = 0 \end{aligned} \tag{17}$$

where space index of x direction is $j = 1, 2, \cdots, J-1$. $k = 0, 1, 2, \cdots, M$ is time index. The variable u_j^k is used to approximate the state variable of temperature $u(k\Delta t, j\Delta x)$ of discrete points, in which $\Delta t = (T/M)$ and $\Delta x = (L/J)$. The state vector and the goal of data assimilation is the same to the first experiment, but the value of N is $N = 6$ in this experiment.

In the numerical experiment, the procedure is the same as the first experiment. The acquisition process of the observations is as follows: the initial state vector is $\boldsymbol{u}_0 = (u_{0,\Delta x}, u_{0,2\Delta x}, ..., u_{0,(N-1)\Delta x}, u_{0,N\Delta x})^T$. The truth initial value is $u_{0,j\Delta x}^{obs} = \sin(j\Delta x\pi)$ $(j = 1, 2, ..., N)$. The time interval is [0, 0.1], the time step and the space grid are $\Delta t = 0.001$ and $\Delta x = 0.25$ respectively. If the background information is not considered, the cost function of discrete form is defined as follows:

$$\hat{J}(\hat{\boldsymbol{u}}) = \frac{1}{2} \sum_{i=1}^{N} \left\| \boldsymbol{u}_i - \boldsymbol{u}_i^{obs} \right\|^2 \tag{18}$$

The initial guess of the initial state vector is $\boldsymbol{u}_0 = (2.0, 2.0, \cdots, 2.0)$. In the process of the iterative estimation for the unknown initial state vector, in order to update the unknown vector $\boldsymbol{u}$ every time, firstly, the dual-number vector $\hat{\boldsymbol{u}}_i = (< u_{i,1}, e_1 >, < u_{i,2}, e_2 >, ..., < u_{i,6}, e_6 >)$ has to be constructed, where $\boldsymbol{e}_j (j = 1, 2, \cdots, 6)$ represents the 6-D unit vector whose jth component is 1, and it is input into the numerical model of the heat conduction equation to make a forward $(0 \to T)$ integration. Secondly, the cost function $\hat{J}(\hat{\boldsymbol{u}})$ is calculated in dual-number space. Thirdly, the real part and the dual part are taken as the value of the cost function J and the gradient vector $\nabla_{\boldsymbol{u}} J$, respectively. Finally, the conjugate gradient algorithm is used to update the value of initial state vector [14]. In order to verify the effectiveness, the state variables from the second time step to the 7th time step are selected as the observation data in the numerical experiment.

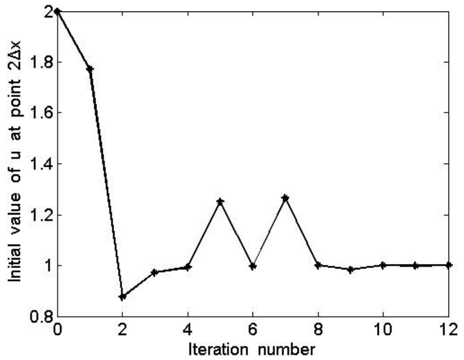

Fig 10. The change of initial state $u_{0,2\Delta x}$ with iteration number.

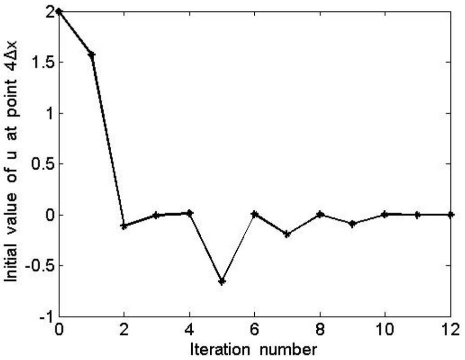

Fig 11. The change of initial state $u_{0,4\Delta x}$ with iteration number.

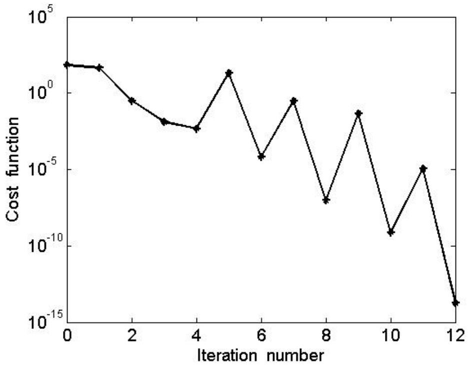

Fig 12. The change of cost function $J(\boldsymbol{u})$ for heat conduction equation.

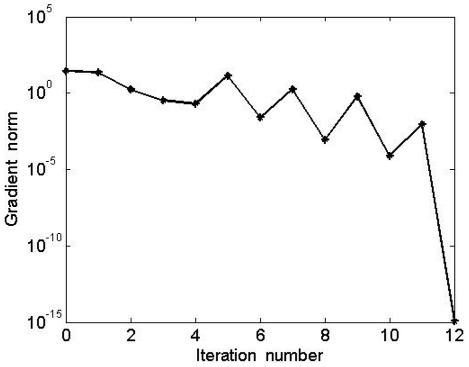

Fig 13. The change of gradient norm $\|\nabla_u J\|$ for heat conduction equation.

Figs 10-13 show the experiment results of data assimilation for heat conduction equation without observation noises. Fig 10 and 11 represent the change of the value for unknown initial state quantities $u_{0,2\Delta x}$ and $u_{0,4\Delta x}$ in the iterative estimation process, respectively. It can be seen from the figures that the iterative value converges to the true value, and the other four points have similar results. In order to avoid redundancy, the results of other four points are not given. Fig 12 shows the change of cost function with iteration number, and Fig 13 shows the change of gradient normal with iteration number. It can be seen from the figure that the cost function achieves convergence after the 12th iteration. The value of the unknown initial state vector $\boldsymbol{u}$ is rounded to the 7th decimal. The results are shown in Table 2. It can be seen from Table 2 that the estimated value is very close to the true value. By analyzing the experiment results, the conclusions are summarized as follows: in case of without background information and only using little observation information, the data assimilation method based on dual-number automatic differentiation can accurately estimate the initial state of the heat conduction equation, and its estimation accuracy reaches $O(10^{-7})$, which verifies the effectiveness of the new data assimilation method based on dual-number automatic differentiation in estimating the unknown initial state for high-dimensional linear physical systems. In addition, with the increasing of observation errors, the accuracy of data assimilation declines, but the iterative estimation results still converge to the true value. When the standard deviation of observation errors is $\sigma_o = 0.2$, the data assimilation results are still close to the true value, and it can be accurate to the first decimal, which illustrates that the new data

Table 2. The results of data assimilation for heat conduction equation under different levels of observation noises.

	$u_{0,\Delta x}$	$u_{0,\Delta 2x}$	$u_{0,\Delta 3x}$	$u_{0,\Delta 4x}$	$u_{0,\Delta 5x}$	$u_{0,\Delta 6x}$	J	number
True value	0.7071068	1.0000000	0.7071068	-0.0000001	-0.7071069	-1.0000000	\	\
$\sigma_o = 0.0$	0.7071068	1.0000000	0.7071068	-0.0000001	-0.7071069	-1.0000000	1.80656E-014	12
$\sigma_o = 0.1$	0.7313267	1.0253142	0.6961767	-0.0277506	-0.7024659	-1.0014807	0.136136834	12
$\sigma_o = 0.2$	0.6891197	0.9900859	0.7204978	-0.0339592	-0.6830015	-1.0387555	0.6611681856	12

assimilation method based on dual-number automatic differentiation is capable of removing noises in the observations when dealing with high-dimensional models.

Conclusion

In order to overcome the shortcomings of the gradient computation for the high-dimensional prediction models in the variational data assimilation when using the adjoint method, a new data assimilation method based on dual-number automatic differentiation is proposed. By using the dual-number automatic differentiation, the process of gradient analysis is transformed to the computation of cost function in dual-number space, which can obtain the value of the gradient vector simply, efficiently and accurately. The important advantages are that the coding of the adjoint model and the reverse integration are not necessary anymore, and the values of cost function and its corresponding gradient vector can be attained simultaneously only by one forward computation in dual-number space. Combining the classical optimization algorithm, the new algorithm and the calculating flow of data assimilation for numerical models are given. Numerical experiments of the new data assimilation method are implemented for a typical nonlinear advection and a parabolic partial differential equation respectively. The results show that the new method can effectively estimate the initial conditions for high-dimensional numerical prediction models and is capable of removing noises. Therefore, the method proposed in this study is a new data assimilation method with strong adaptability.

Author Contributions

Conceived and designed the experiment:GW XC. Performed the experiments:GW. Analyzed the data:GW XC. Contributed reagents/materials/analysis tools:XC JS. Wrote the paper:GW XC JS

References

1. Guan YH, Zhou GQ, Lu WS, Chen JP. Theory development and application of data assimilation methods. Meteorology and Disaster Reduction Research. 2007;30(4):938–950. doi: 10.3969/j.issn.1007-9033.2007.04.001

2. Li H, Xu JP. Development of data assimilation and its application in ocean science. Marine Science Bulletin. 2011;30(4):463-472. doi: 10.3969/j.issn.1001-6392.2011.04.018

3. Zhu GF. Understanding of the fundamental concept of atmospheric data assimilation. Meteorological Monthly. 2015;41(4):456-463. doi: 10.7519/j.issn.1000-0526.2015.04.008

4. She ZQ, Tang YM, Gao YQ. The theoretical framework of the ensemble-based data assimilation method and its prospect in oceanic data assimilation. Haiyang Xuebao. 2016;0(3):1-14. doi: 10.3969/j.issn.0253-4193.2016.03.001

5. Evensen G. Sequential data assimilation with a nonlinear quasi-geostrophic model using Monte Carlo methods to forecast error statistics. Journal of Geophysical Research Atmospheres. 1994;99(C5):10143-10162. doi: 10.1029/94JC00572

6. Thepaut JN, Courtier P. Four-dimensional data assimilation using the adjoint of a multiple primitive-equation mode. Quarterly Journal of the Royal Meteorological Society. 1991;117(502):1225-1254. doi: 10.1002/qj.49711750206

7. Rabier F, Mahfouf JF, Jarvinen H, Klinker E, Simmons A. The ECMWF operational implementation of four-dimensional variational assimilation. II: Experimental results with improved physics. Quarterly Journal of the Royal Meteorological Society. 2000;126(564):1171-1190 doi:10.1002/qj.49712656416

8. Cao XQ, Huang SX, Du Hd. The new method of modeling horizontal error functions in variational assimilation with orthogonal wavelet. Acta Physica Sinica. 2008;57(3):1984-1989. doi: 10.3321/j.issn:1000-3290.2008.03.115

9. Cao XQ, Song JQ, Zhang WM, Zhao YL, Liu BN. A new data assimilation method using complex-variable differentiation. Acta Physica Sinica. 2013;62(17):170504-170504. doi: 10.7498/aps.62.170504

10. Zhang WM, Cao XQ, Song JQ. Design and implementation of four-dimensional variational data assimilation system constrained by the global spectral model. Acta Physica Sinica. 2012;61(24):249202-249202. doi: 10.7498/aps.61.249202.

11. Giering R, Kaminski T. Recipes for adjoint code construction. Acm Transactions on Mathematical Software. 1998;24(4):437-474. doi: 10.1145/293686.293695

12. Cheng Q, Zhang HB, Wang B. Algorithms of automatic differentiation. Mathematical Numerica Sinica. 2009;31(1):15-36. doi: 10.3321/j.issn:0254-7791.2009.01.002

13. Lyness JN; Moler CB. Numerical differentiation of analytic functions. Siam Journal on Numerical Analysis. 1967;4(2):202-210. doi: 10.1137/0704019

14. Cao XQ, Song JQ, Zhang WM, Zhao YL, Liu BN. A new data assimilation method using complex-variable differentiation. Acta Physica Sinica. 2013;62(17):170504-170504. doi: 10.7498/aps.62.170504

15. Cao XQ, Huang QB, Liu BN, Zhu MB, Yu Y. A new data assimilation method based on dual-number theory. Acta Physica Sinica. 2015;64(13):8-19. doi: 10.7498/aps.64.130502

16. He JH; Lee EWM. A constrained variational principle for heat conduction. Physics Letters A. 2009;373(31):2614-2615. doi: 10.1016/j.physleta.2009.05.039

17. He JH; Wu XH. Variational iteration method: New development and applications. Computers & Mathematics with Applications. 2007;54(7-8):881-894. doi: 10.1016/j.camwa.2006.12.083

18. Brodsky V; Shoham M. Dual numbers representation of rigid body dynamics. Mechanism & Machine Theory. 1999;34(5):693-718. doi: 10.1016/S0094-114X(98)00049-4

19. Wang JY, Liang HC, Sun ZW. Dual number-based relative coupled dynamics control. Journal of Astronautics. 2010;31(7):1711-1717. doi: 10.3873/j.issn.1000-1328.2010.07.003

20. Spall RE; Yu W. Imbedded dual-number automatic differentiation for Computational Fluid Dynamics sensitivity analysis. Journal of Fluids Engineering. 2012;135(1):279-284. doi: 10.1115/1.4023074

21. Yu W; Blair M. DNAD, a simple tool for automatic differentiation of Fortran codes using dual numbers. Computer Physics Communications. 2013;184(5):1446-1452. doi: 10.1016/j.cpc.2012.12.025

22. Yang XZ, Zhang H, Lu P, Ji ZZ. Stability analysis for difference schemes of nonlinear advection equations. Journal of China Electric Power University. 1999;26(4):84-89.

23. Zhou BB. A kind of explicit finite difference schemes with better stability for advective equations. Scientia Atmospherica Sinica. 1995;19(2):252-256. doi: 10.3878/j.issn.1006-9895.1995.02.15

24. Isakov V; Kindermann S. Identification of the diffusion coefficient in a one-dimensional parabolic equation. Inverse Problems. 2000;16(3):665-68010. doi: 1088/0266-5611/16/3/309
