# Supplementary material for: A new data assimilation method for high-dimensional models
Source: PLoS One. 2018 Feb 8;13(2):e0191714. doi: 10.1371/journal.pone.0191714 (PMC5805242; doi:10.1371/journal.pone.0191714)
Supplement: S7 File — This file Includes all the result and figures used in the manuscript. (ZIP) [file pone.0191714.s007.zip › minor revision/figures/flow-eps-converted-to.pdf]

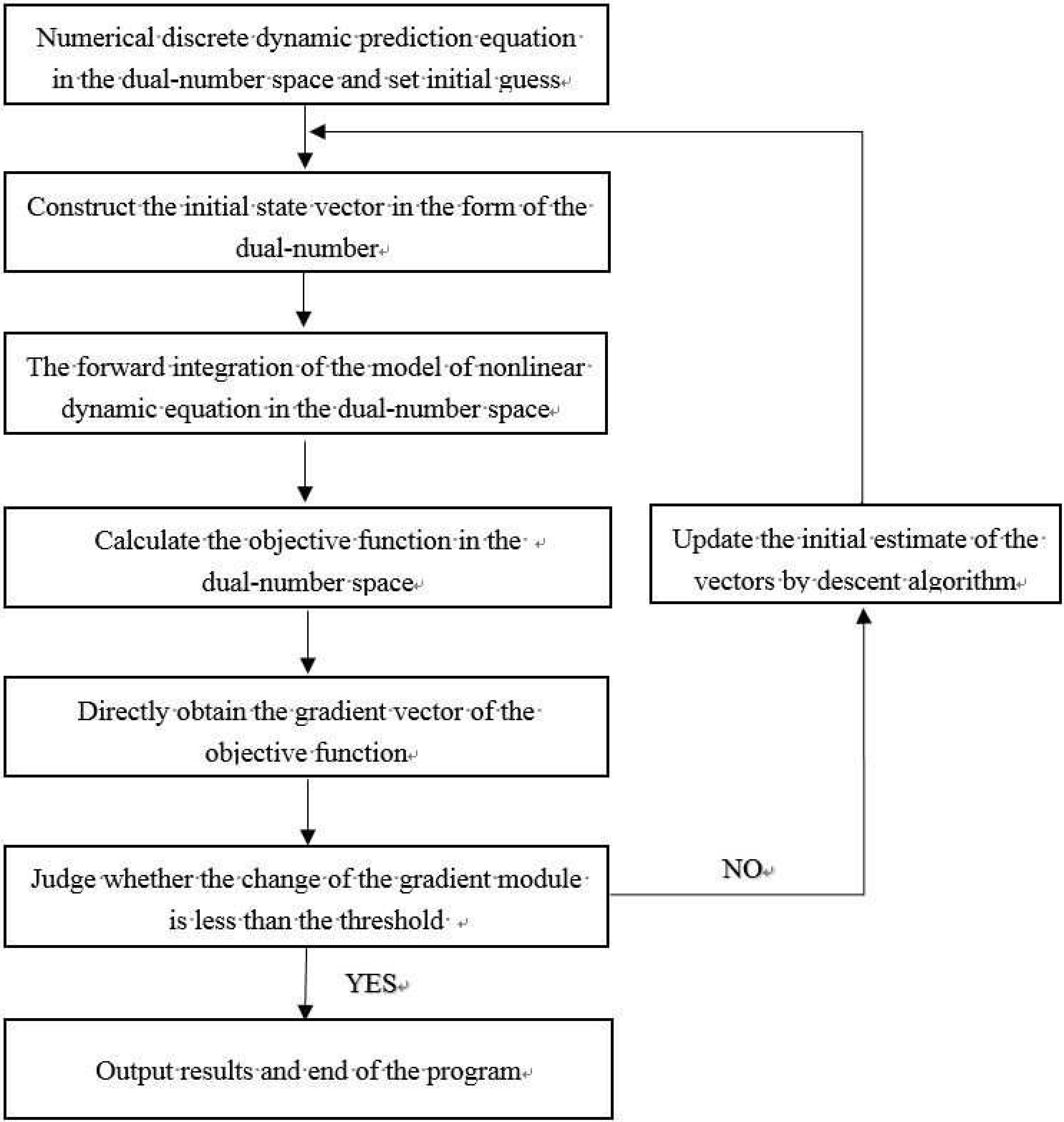

Numerical discrete dynamic prediction equation in the dual-number space and set initial guess
Construct the initial state vector in the form of the dual-number
The forward integration of the model of nonlinear dynamic equation in the dual-number space
Calculate the objective function in the dual-number space
Directly obtain the gradient vector of the objective function
Judge whether the change of the gradient module is less than the threshold
YES
Output results and end of the program
NO
Update the initial estimate of the vectors by descent algorithm
