# Supplementary figures and images for: A new data assimilation method for high-dimensional models
Source: PLoS One. 2018 Feb 8;13(2):e0191714. doi: 10.1371/journal.pone.0191714 (PMC5805242; doi:10.1371/journal.pone.0191714)

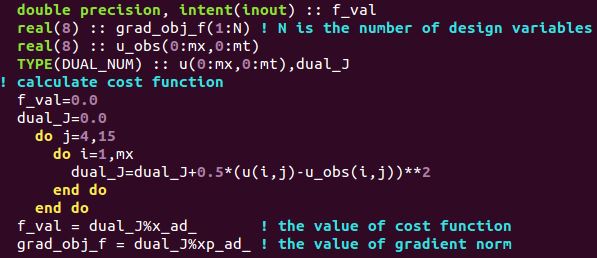

Supplement: S7 File — This file Includes all the result and figures used in the manuscript. (ZIP) [file pone.0191714.s007.zip › minor revision/figures/cost.JPG]

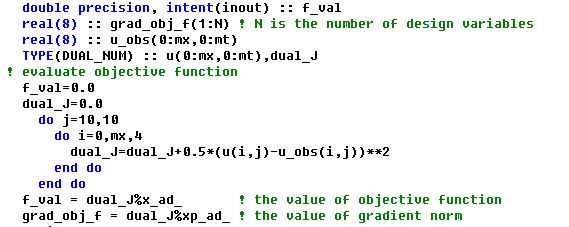

Supplement: S7 File — This file Includes all the result and figures used in the manuscript. (ZIP) [file pone.0191714.s007.zip › minor revision/figures/cost1.JPG]

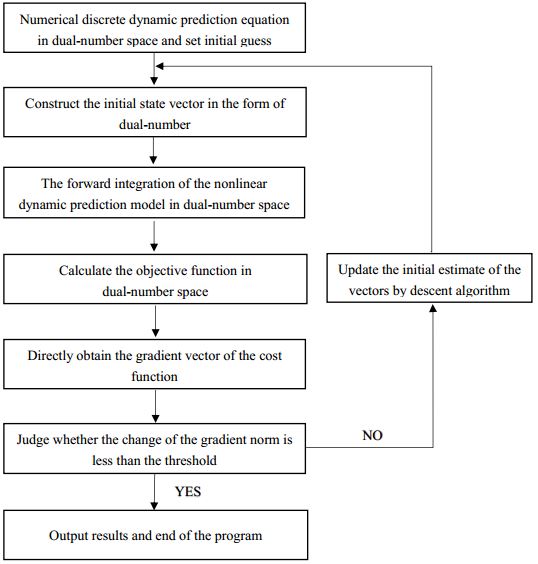

Supplement: S7 File — This file Includes all the result and figures used in the manuscript. (ZIP) [file pone.0191714.s007.zip › minor revision/figures/fl.jpg]

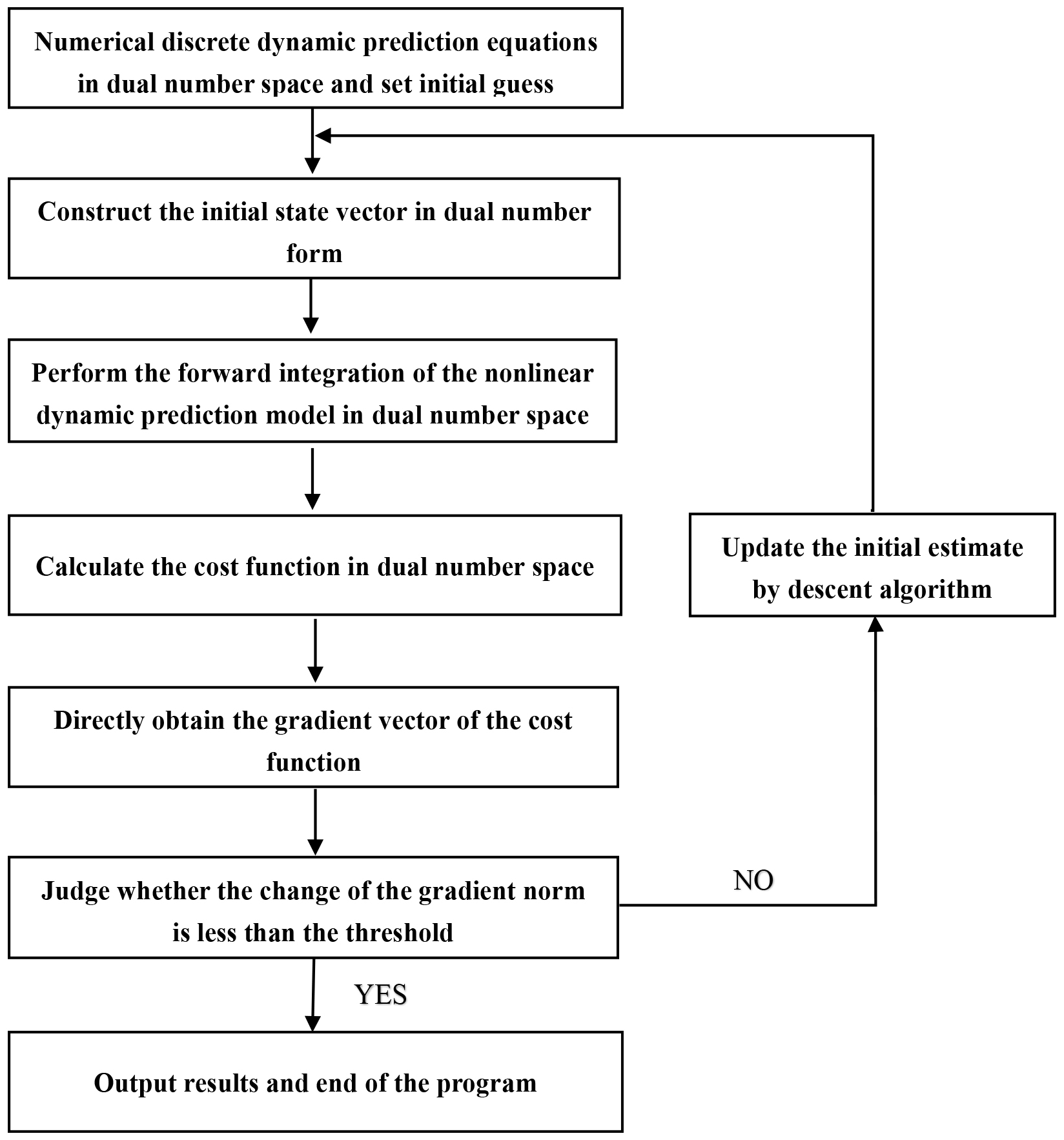

Supplement: S7 File — This file Includes all the result and figures used in the manuscript. (ZIP) [file pone.0191714.s007.zip › minor revision/figures/flow.jpg]

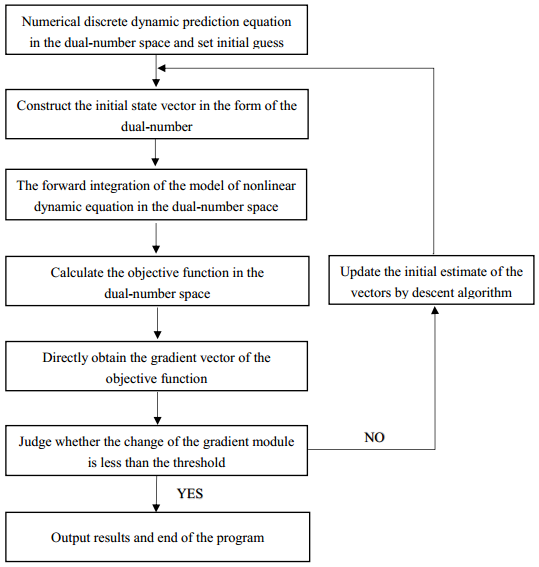

Supplement: S7 File — This file Includes all the result and figures used in the manuscript. (ZIP) [file pone.0191714.s007.zip › minor revision/figures/flow.PNG]

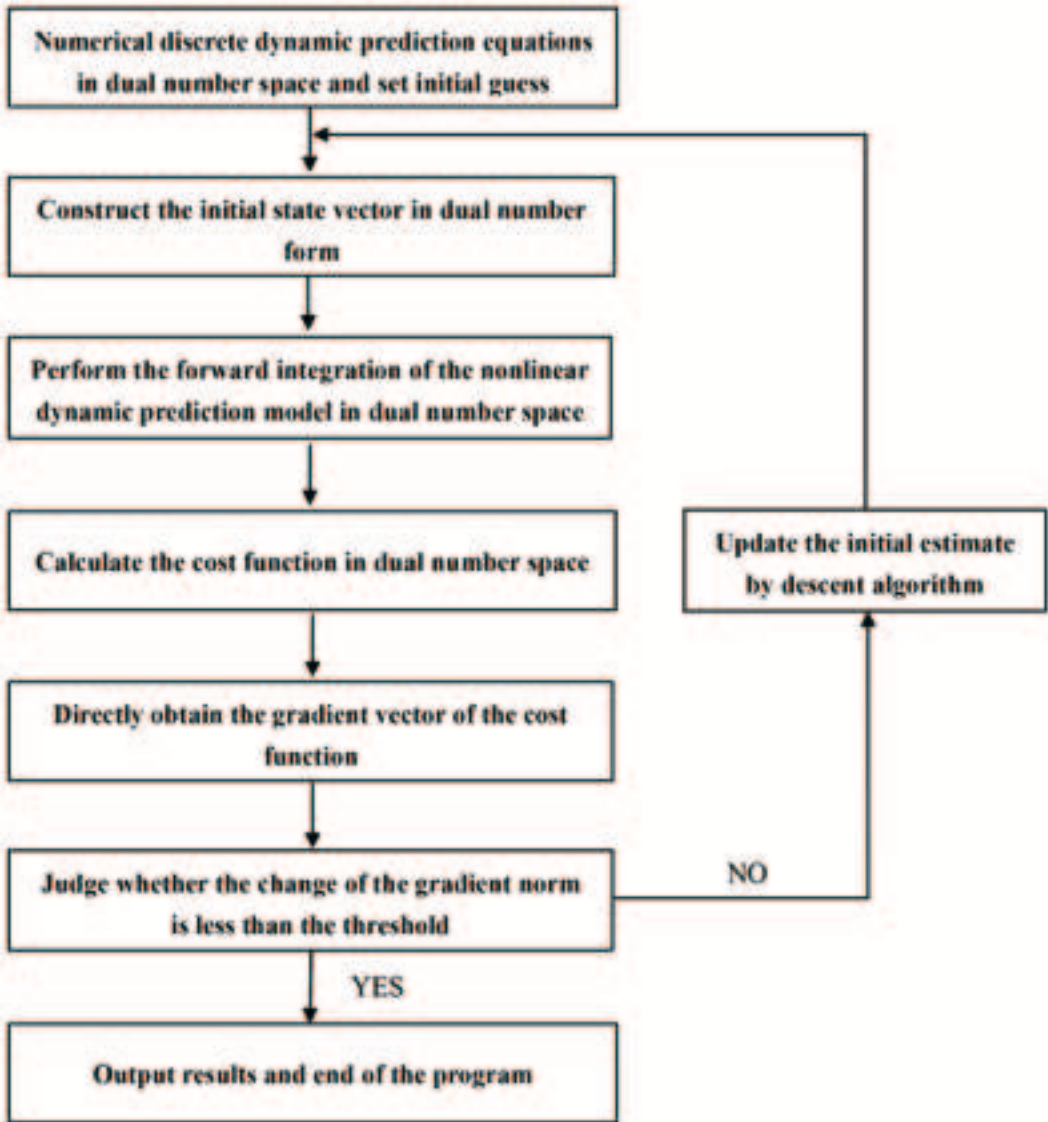

Supplement: S7 File — This file Includes all the result and figures used in the manuscript. (ZIP) [file pone.0191714.s007.zip › minor revision/figures/flow1-eps-converted-to.pdf]

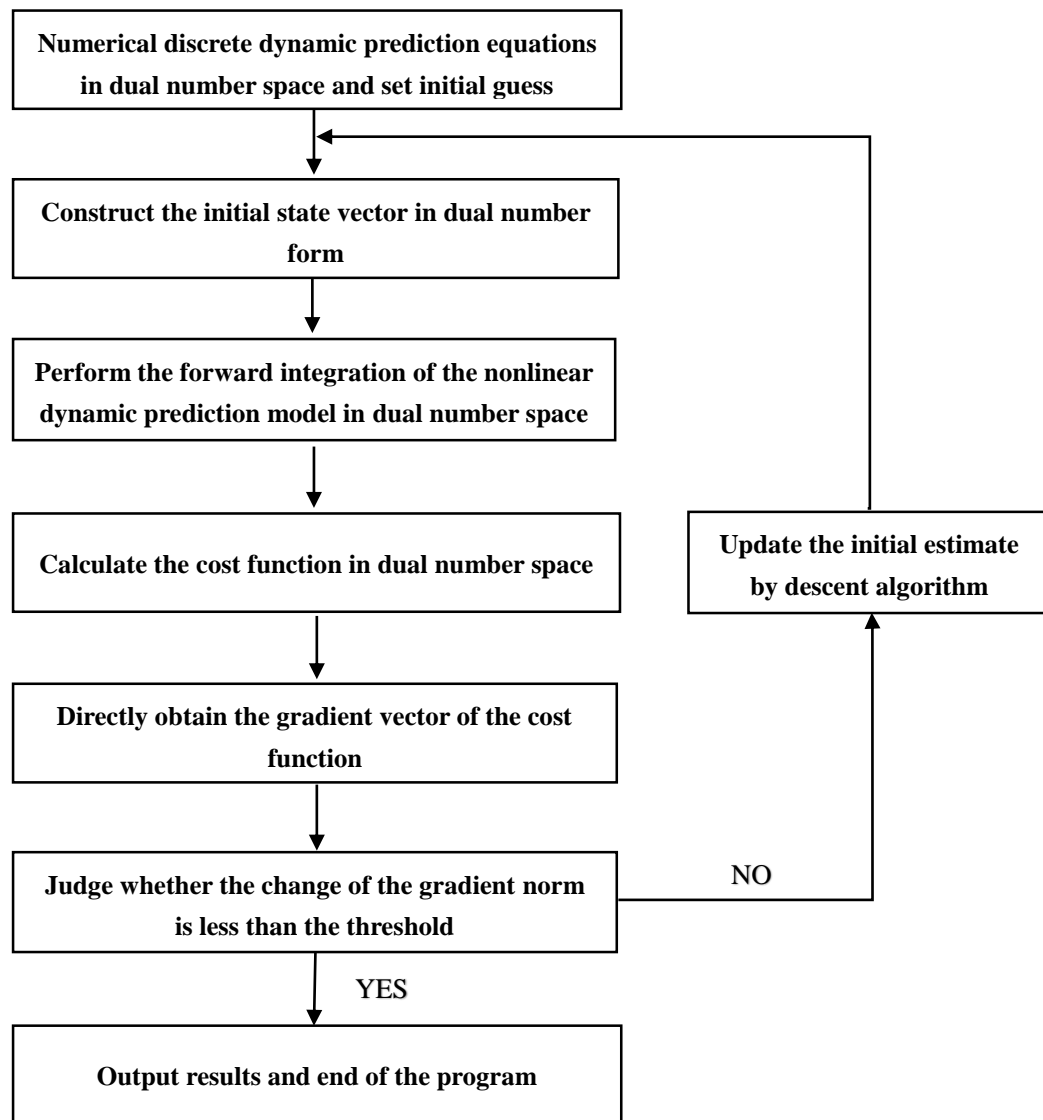

Supplement: S7 File — This file Includes all the result and figures used in the manuscript. (ZIP) [file pone.0191714.s007.zip › minor revision/figures/flow1.pdf]

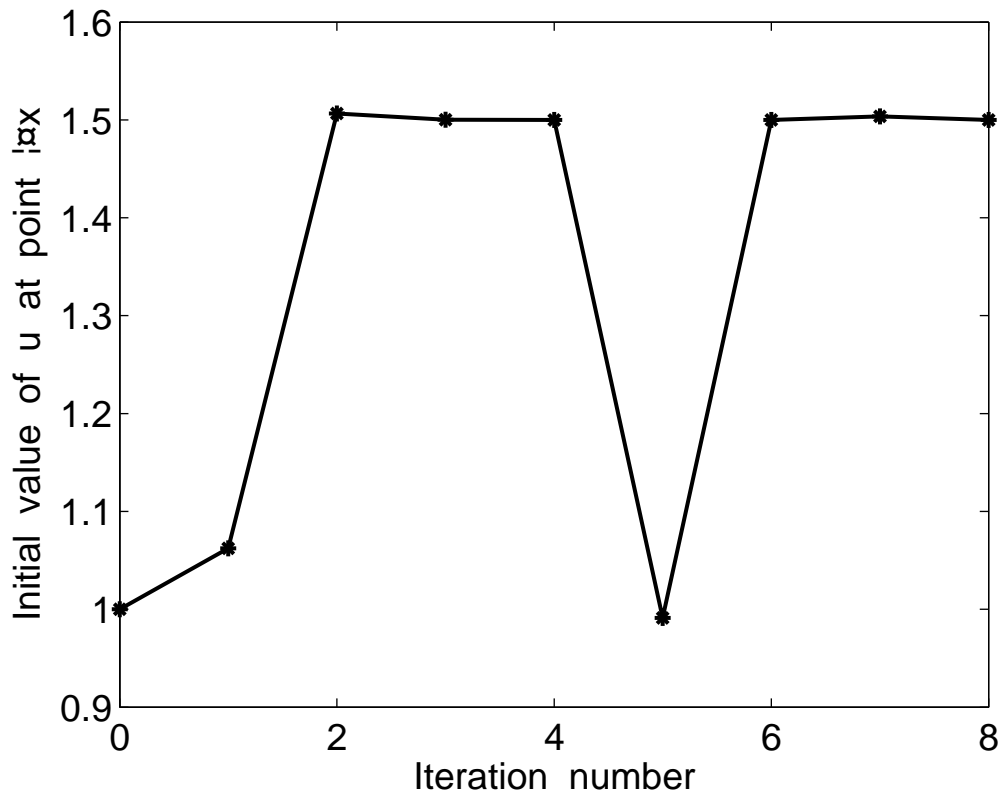

Supplement: S7 File — This file Includes all the result and figures used in the manuscript. (ZIP) [file pone.0191714.s007.zip › minor revision/figures/linear/1-eps-converted-to.pdf]

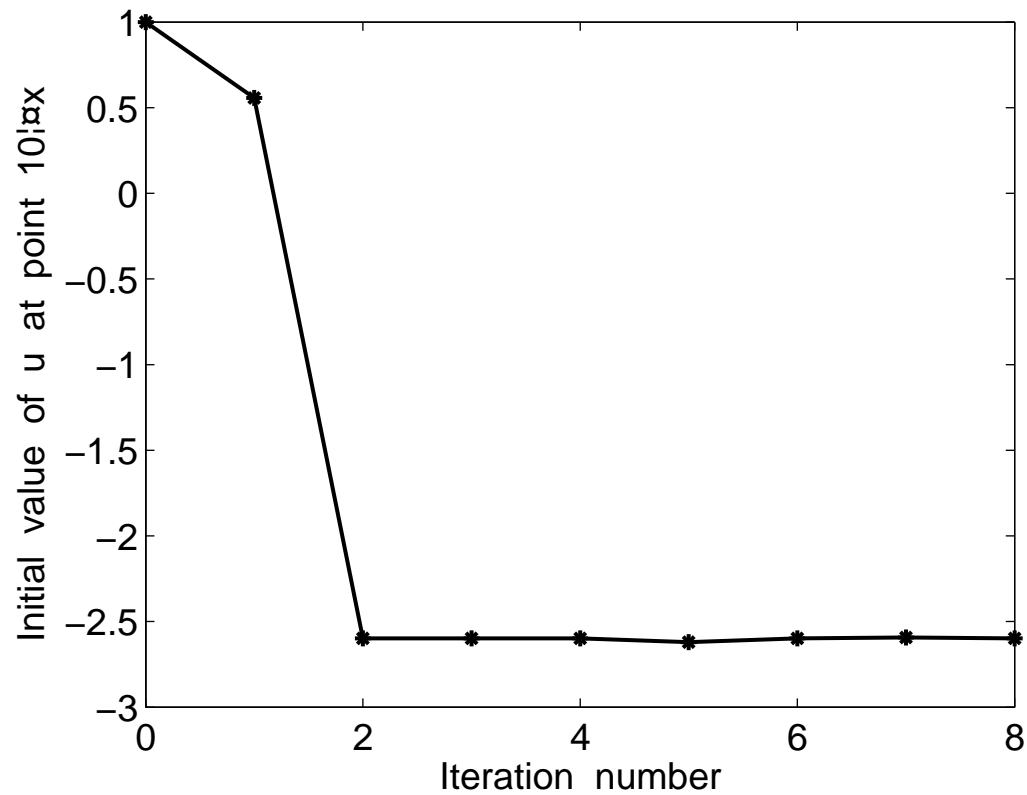

Supplement: S7 File — This file Includes all the result and figures used in the manuscript. (ZIP) [file pone.0191714.s007.zip › minor revision/figures/linear/10-eps-converted-to.pdf]

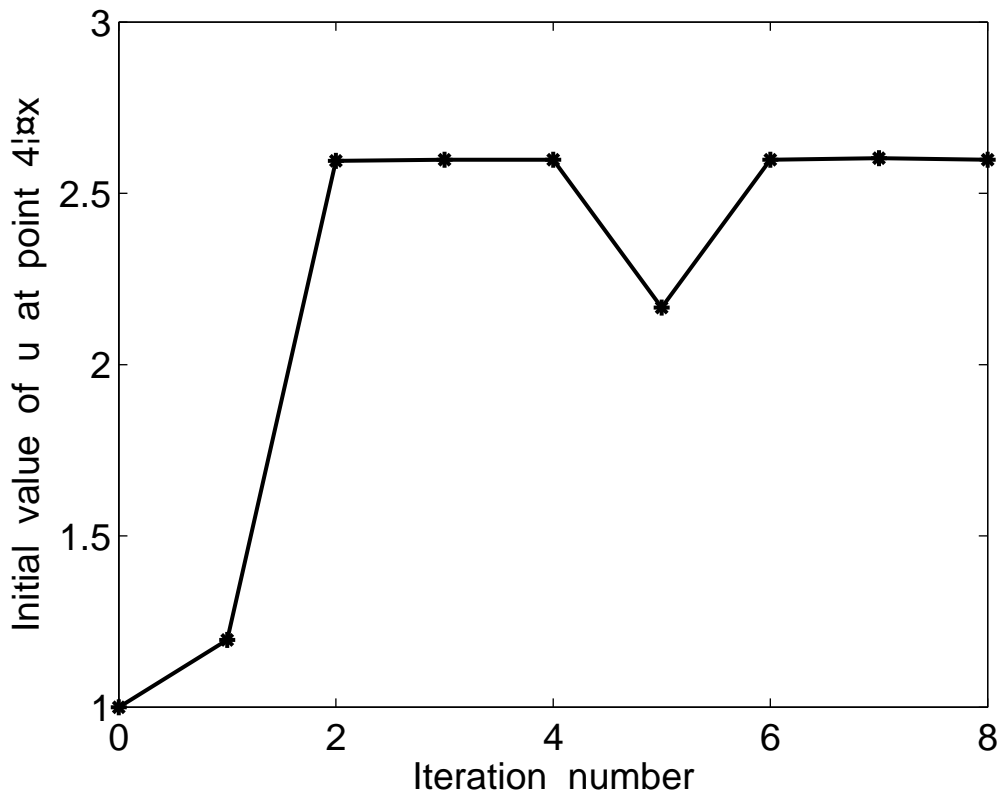

Supplement: S7 File — This file Includes all the result and figures used in the manuscript. (ZIP) [file pone.0191714.s007.zip › minor revision/figures/linear/4-eps-converted-to.pdf]

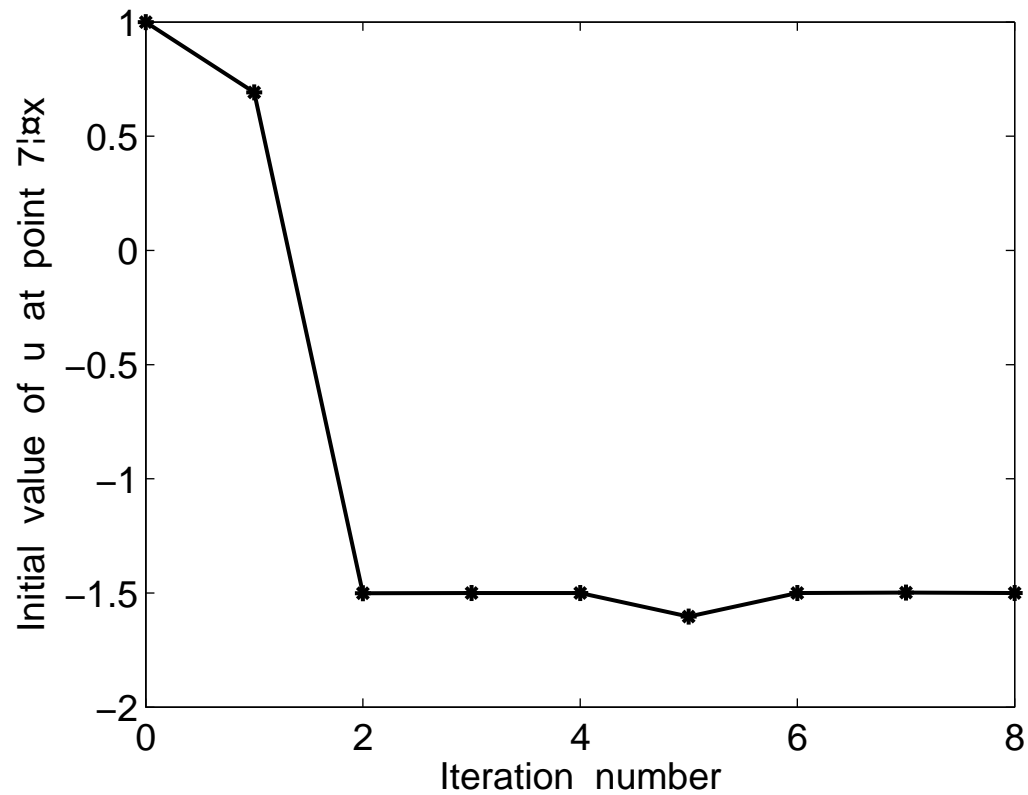

Supplement: S7 File — This file Includes all the result and figures used in the manuscript. (ZIP) [file pone.0191714.s007.zip › minor revision/figures/linear/7-eps-converted-to.pdf]

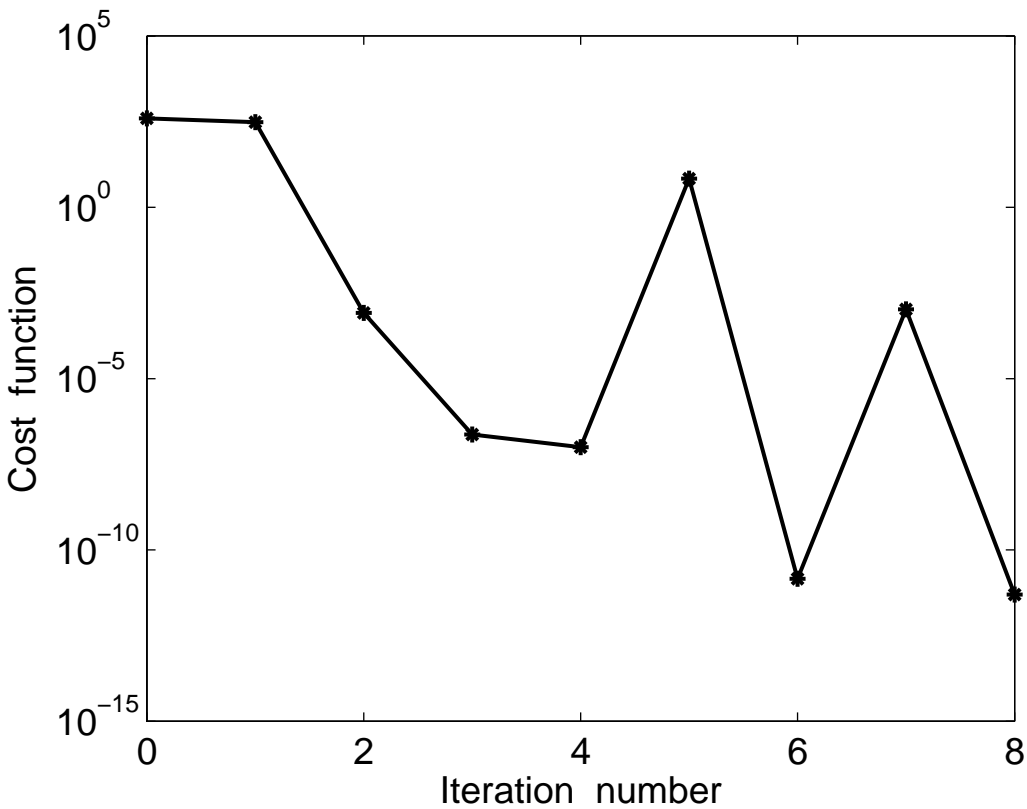

Supplement: S7 File — This file Includes all the result and figures used in the manuscript. (ZIP) [file pone.0191714.s007.zip › minor revision/figures/linear/cost-eps-converted-to.pdf]

Gradient norm

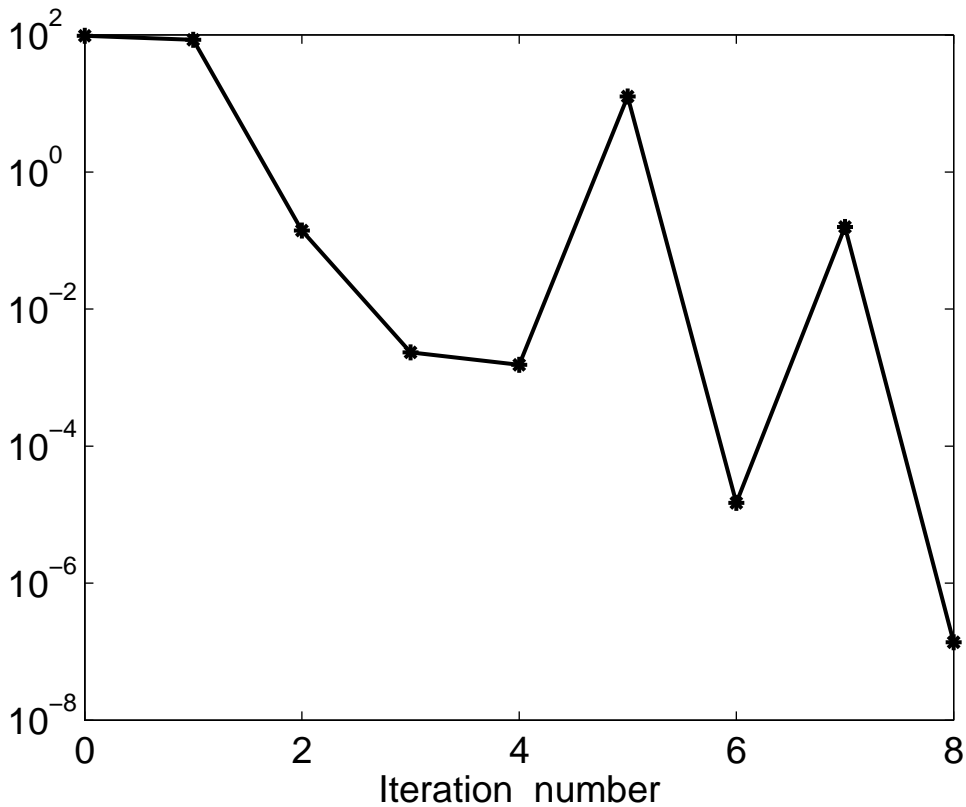

Supplement: S7 File — This file Includes all the result and figures used in the manuscript. (ZIP) [file pone.0191714.s007.zip › minor revision/figures/linear/norm-eps-converted-to.pdf]

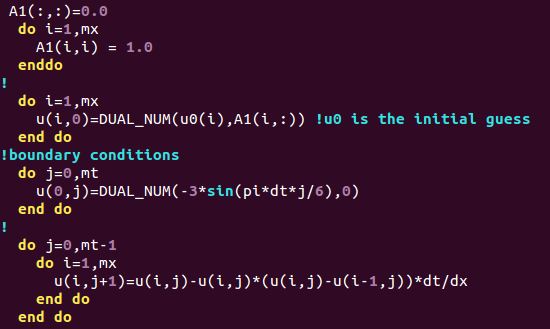

Supplement: S7 File — This file Includes all the result and figures used in the manuscript. (ZIP) [file pone.0191714.s007.zip › minor revision/figures/model.JPG]

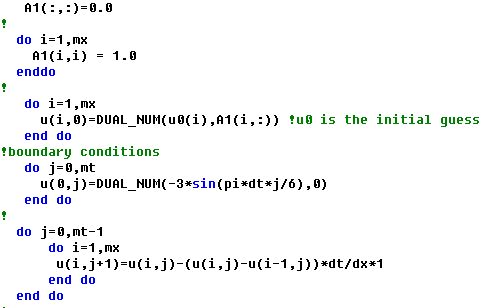

Supplement: S7 File — This file Includes all the result and figures used in the manuscript. (ZIP) [file pone.0191714.s007.zip › minor revision/figures/model1.JPG]

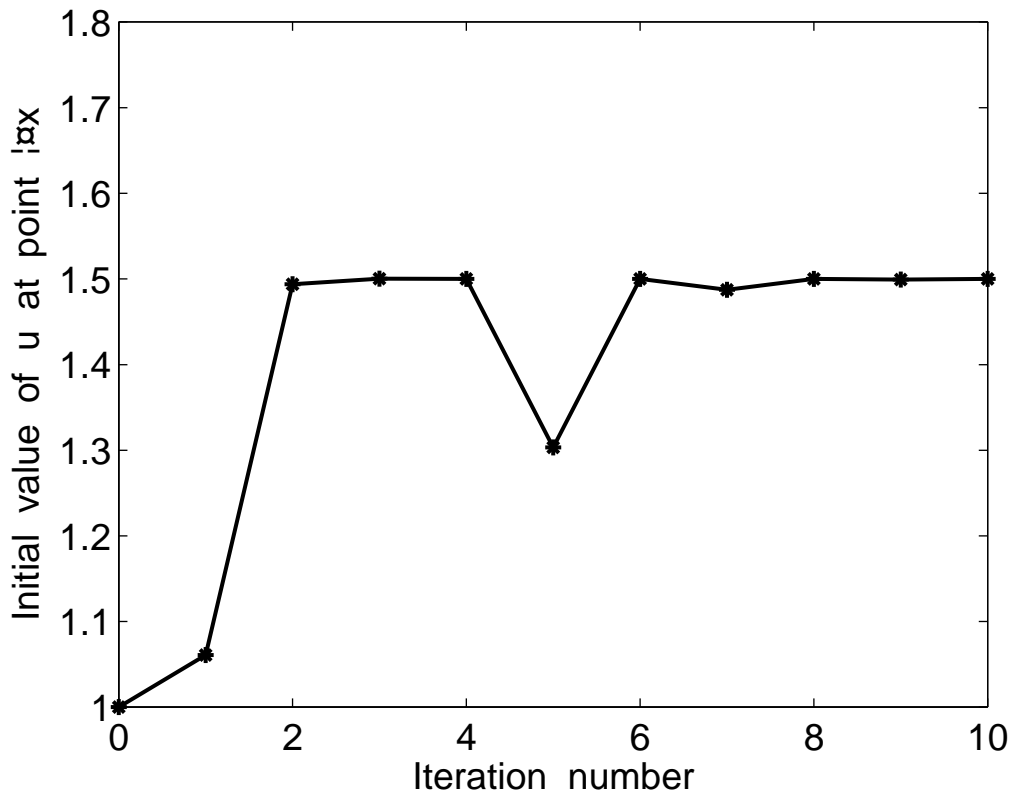

Supplement: S7 File — This file Includes all the result and figures used in the manuscript. (ZIP) [file pone.0191714.s007.zip › minor revision/figures/nolinear/1-eps-converted-to.pdf]

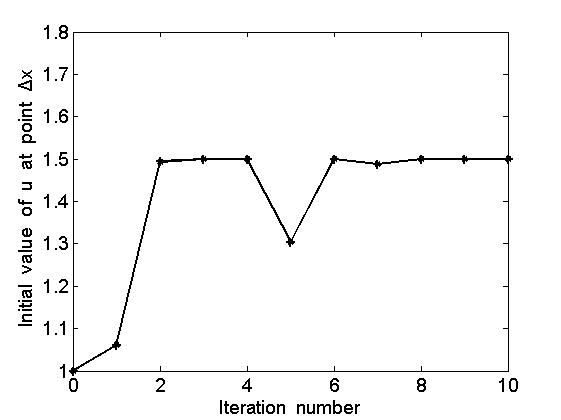

Supplement: S7 File — This file Includes all the result and figures used in the manuscript. (ZIP) [file pone.0191714.s007.zip › minor revision/figures/nolinear/1.jpg]

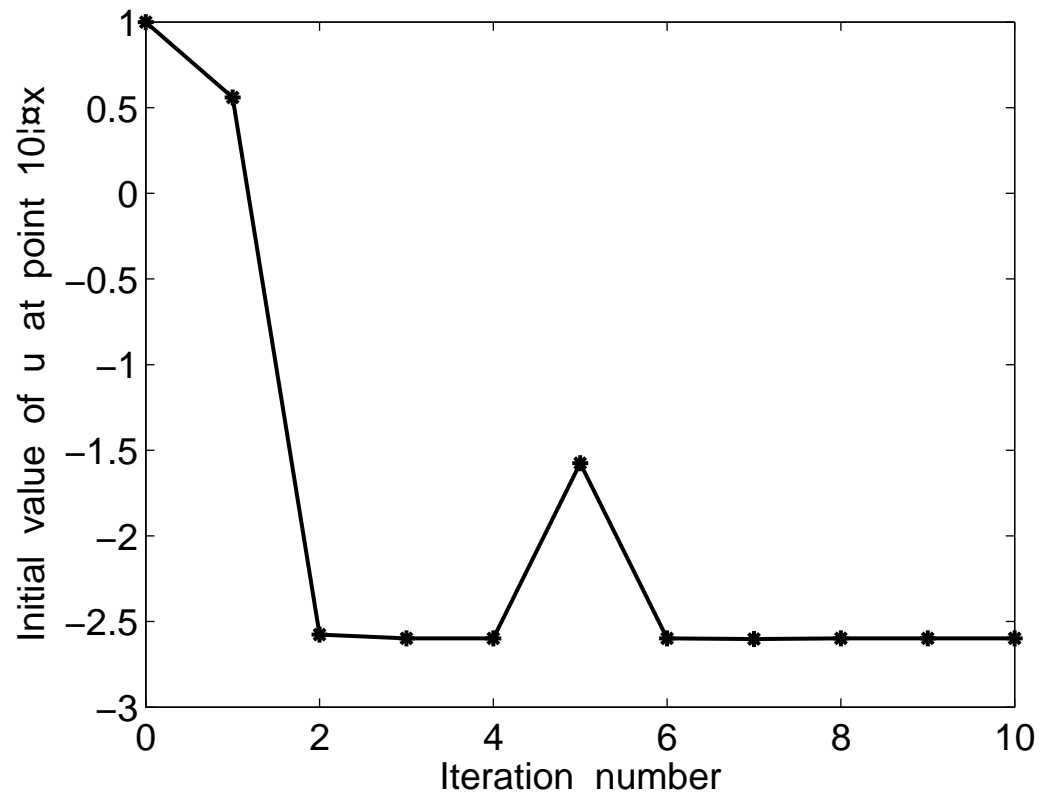

Supplement: S7 File — This file Includes all the result and figures used in the manuscript. (ZIP) [file pone.0191714.s007.zip › minor revision/figures/nolinear/10-eps-converted-to.pdf]

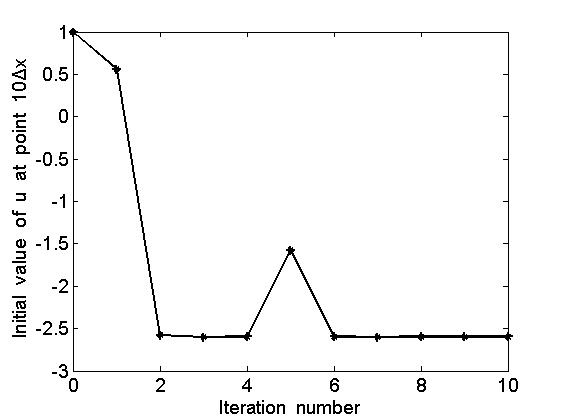

Supplement: S7 File — This file Includes all the result and figures used in the manuscript. (ZIP) [file pone.0191714.s007.zip › minor revision/figures/nolinear/10.jpg]

(a)

Initial value of  $u$  at point  $Dx$

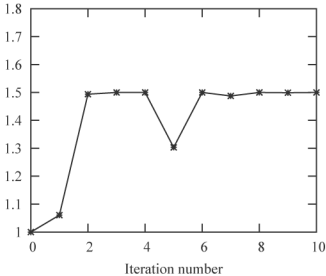

Supplement: S7 File — This file Includes all the result and figures used in the manuscript. (ZIP) [file pone.0191714.s007.zip › minor revision/figures/nolinear/11-eps-converted-to.pdf]

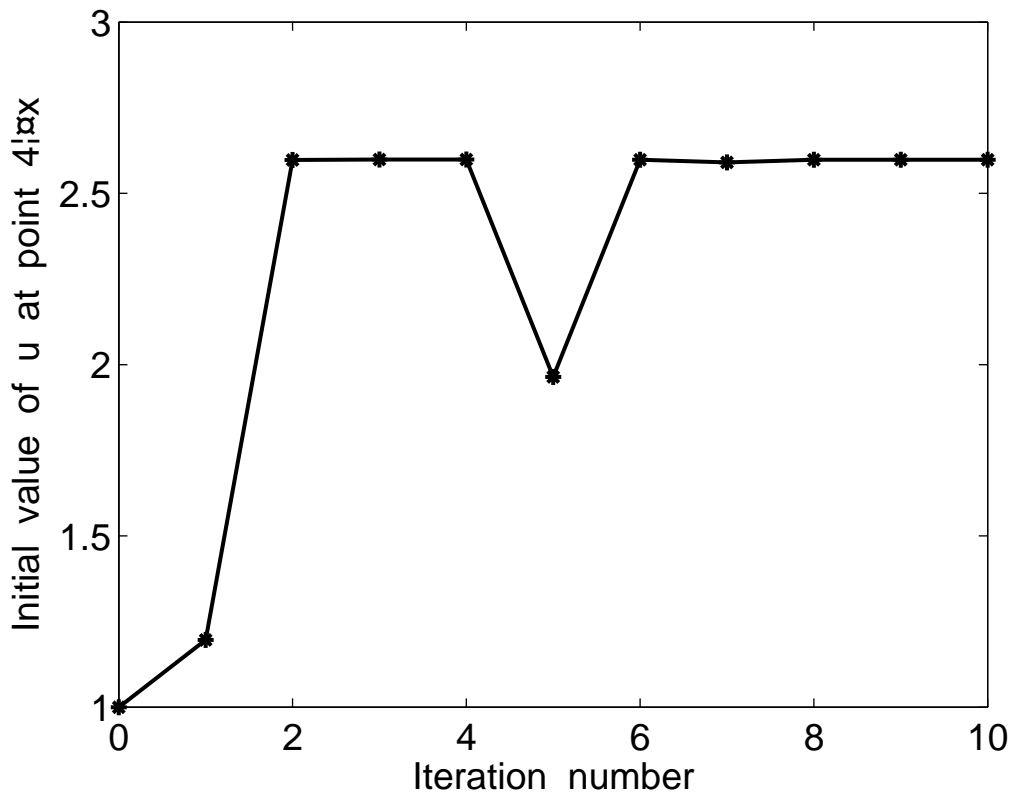

Supplement: S7 File — This file Includes all the result and figures used in the manuscript. (ZIP) [file pone.0191714.s007.zip › minor revision/figures/nolinear/4-eps-converted-to.pdf]

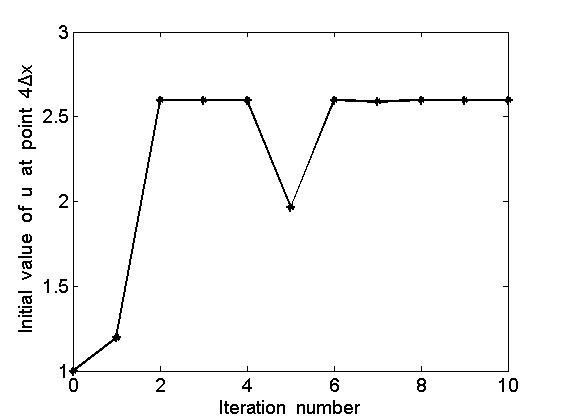

Supplement: S7 File — This file Includes all the result and figures used in the manuscript. (ZIP) [file pone.0191714.s007.zip › minor revision/figures/nolinear/4.jpg]

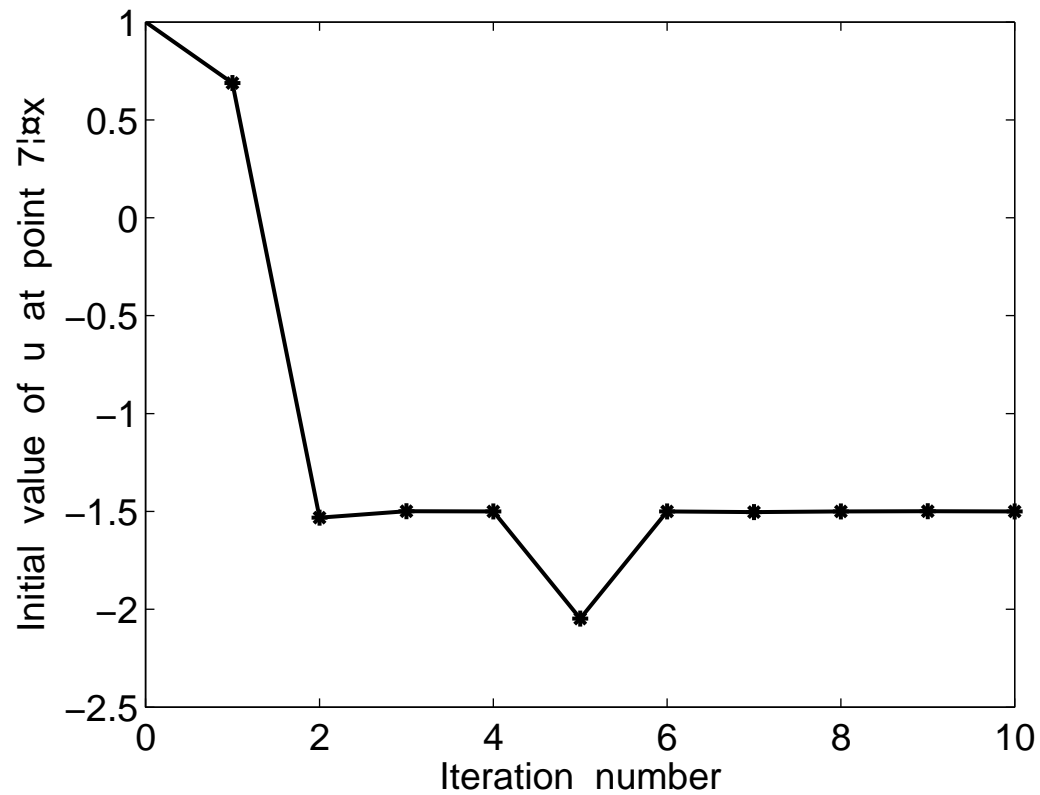

Supplement: S7 File — This file Includes all the result and figures used in the manuscript. (ZIP) [file pone.0191714.s007.zip › minor revision/figures/nolinear/7-eps-converted-to.pdf]

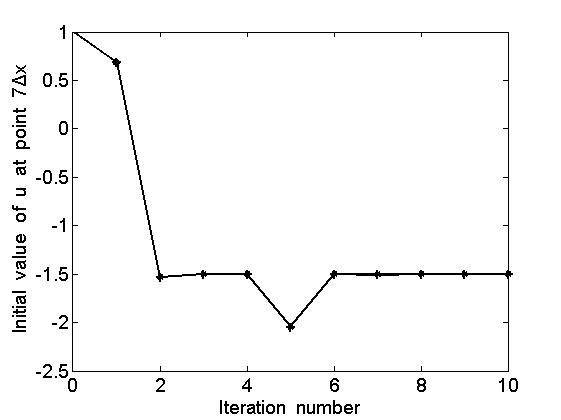

Supplement: S7 File — This file Includes all the result and figures used in the manuscript. (ZIP) [file pone.0191714.s007.zip › minor revision/figures/nolinear/7.jpg]

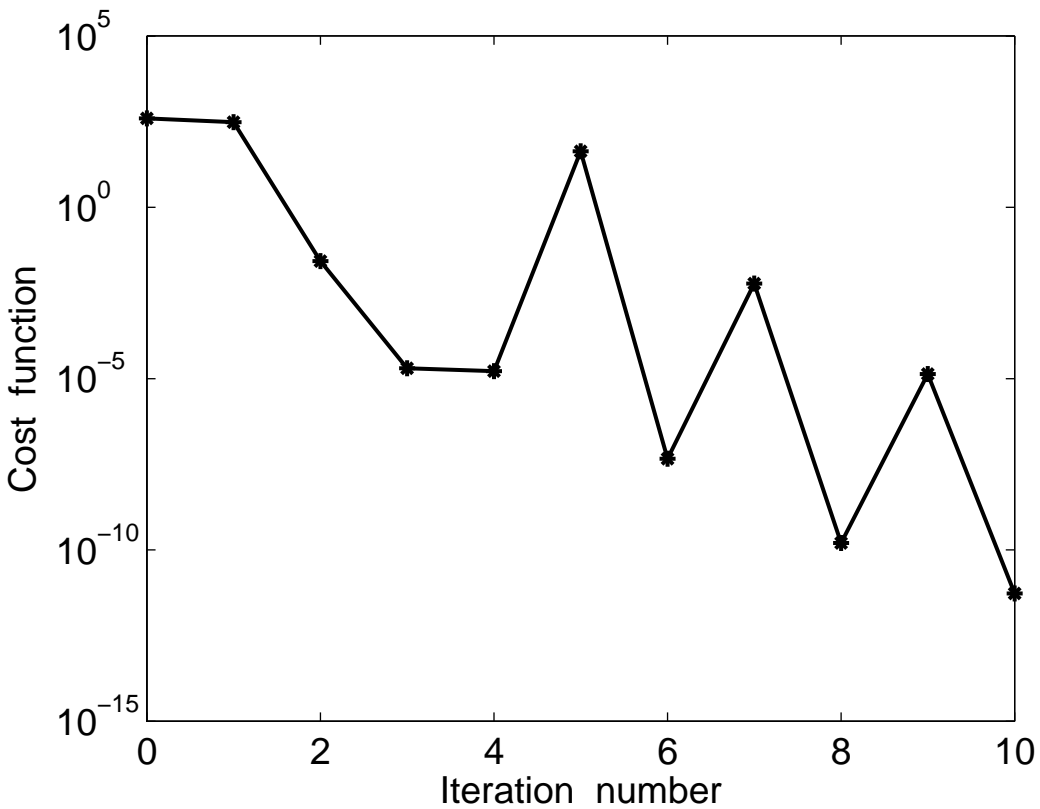

Supplement: S7 File — This file Includes all the result and figures used in the manuscript. (ZIP) [file pone.0191714.s007.zip › minor revision/figures/nolinear/cost-eps-converted-to.pdf]

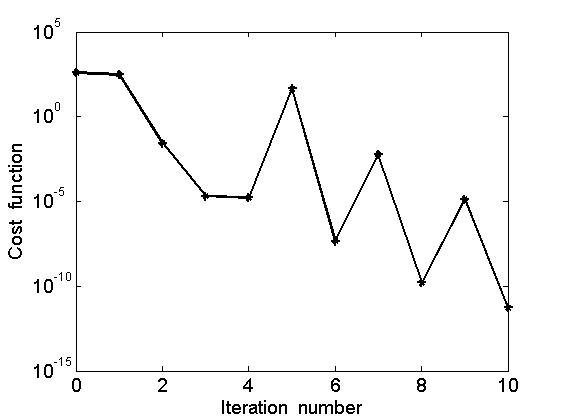

Supplement: S7 File — This file Includes all the result and figures used in the manuscript. (ZIP) [file pone.0191714.s007.zip › minor revision/figures/nolinear/cost.jpg]

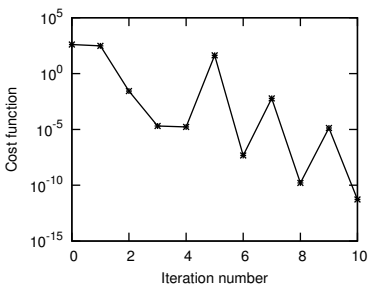

Supplement: S7 File — This file Includes all the result and figures used in the manuscript. (ZIP) [file pone.0191714.s007.zip › minor revision/figures/nolinear/Costfunction-eps-converted-to.pdf]

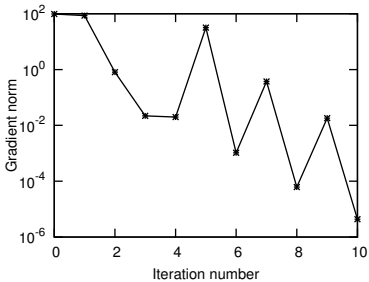

Supplement: S7 File — This file Includes all the result and figures used in the manuscript. (ZIP) [file pone.0191714.s007.zip › minor revision/figures/nolinear/Gradientnorm-eps-converted-to.pdf]

Initial value of  $u$  at point  $\Delta x$

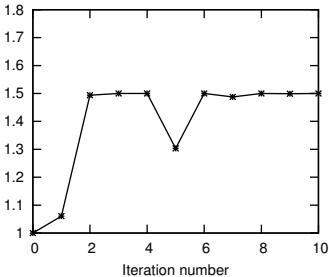

Supplement: S7 File — This file Includes all the result and figures used in the manuscript. (ZIP) [file pone.0191714.s007.zip › minor revision/figures/nolinear/nonline1-eps-converted-to.pdf]

Initial value of  $u$  at point  $10\Delta x$

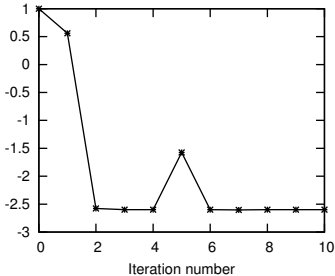

Supplement: S7 File — This file Includes all the result and figures used in the manuscript. (ZIP) [file pone.0191714.s007.zip › minor revision/figures/nolinear/nonline10-eps-converted-to.pdf]

Initial value of  $u$  at point  $4\Delta x$

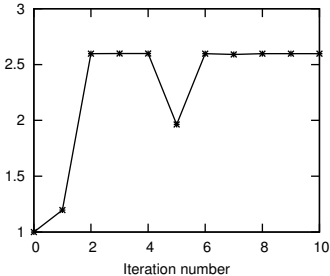

Supplement: S7 File — This file Includes all the result and figures used in the manuscript. (ZIP) [file pone.0191714.s007.zip › minor revision/figures/nolinear/nonline4-eps-converted-to.pdf]

Initial value of  $u$  at point  $7\Delta x$

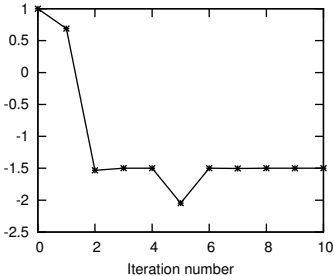

Supplement: S7 File — This file Includes all the result and figures used in the manuscript. (ZIP) [file pone.0191714.s007.zip › minor revision/figures/nolinear/nonline7-eps-converted-to.pdf]

Gradient norm

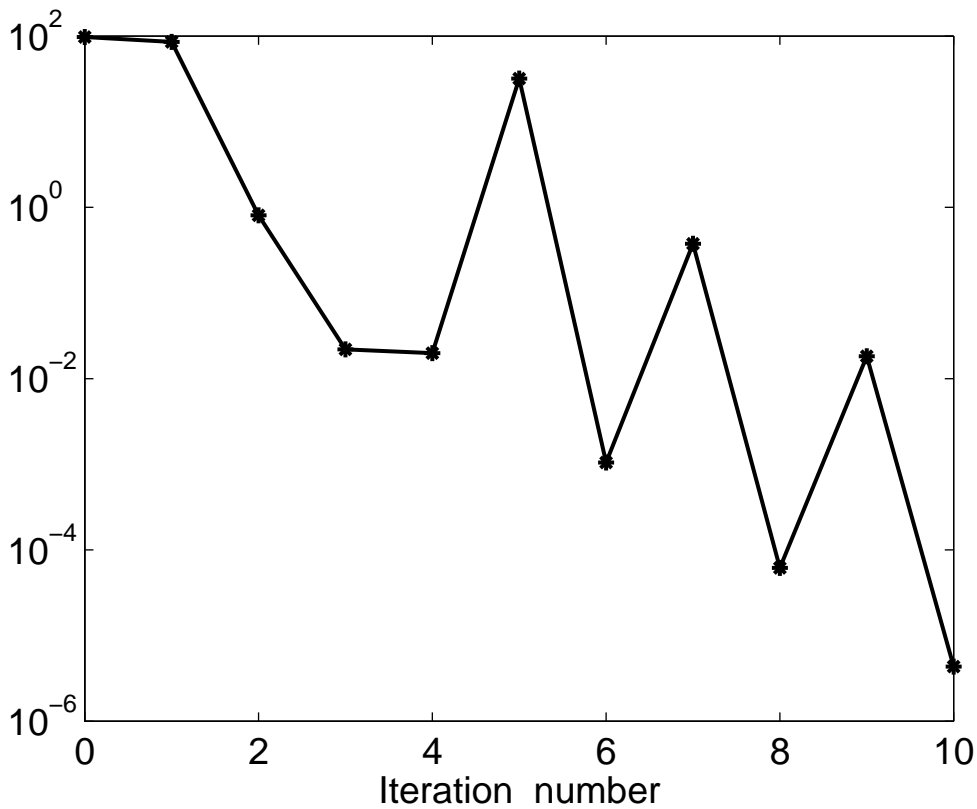

Supplement: S7 File — This file Includes all the result and figures used in the manuscript. (ZIP) [file pone.0191714.s007.zip › minor revision/figures/nolinear/norm-eps-converted-to.pdf]

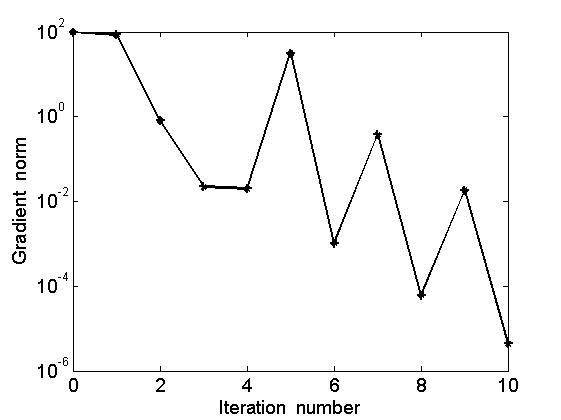

Supplement: S7 File — This file Includes all the result and figures used in the manuscript. (ZIP) [file pone.0191714.s007.zip › minor revision/figures/nolinear/norm.jpg]

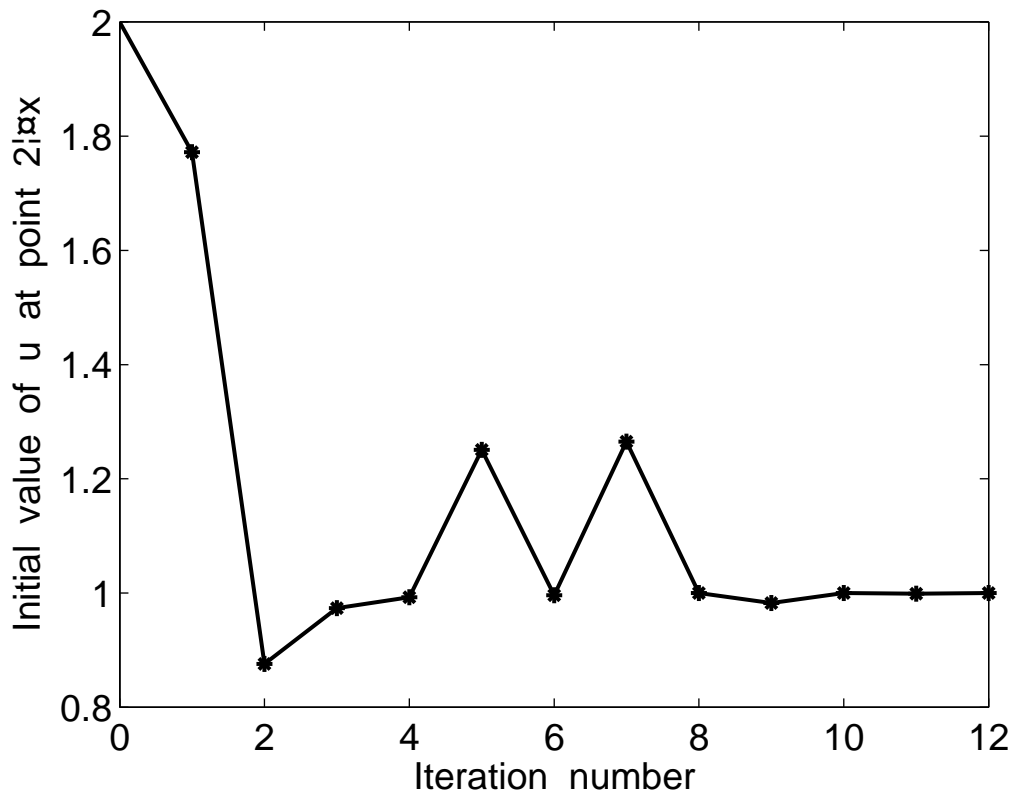

Supplement: S7 File — This file Includes all the result and figures used in the manuscript. (ZIP) [file pone.0191714.s007.zip › minor revision/figures/parabolic/2-eps-converted-to.pdf]

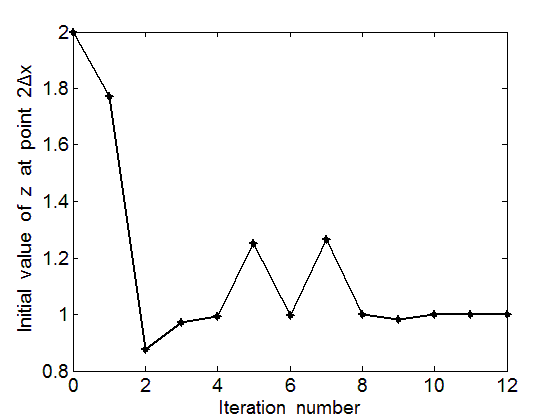

Supplement: S7 File — This file Includes all the result and figures used in the manuscript. (ZIP) [file pone.0191714.s007.zip › minor revision/figures/parabolic/2.bmp]

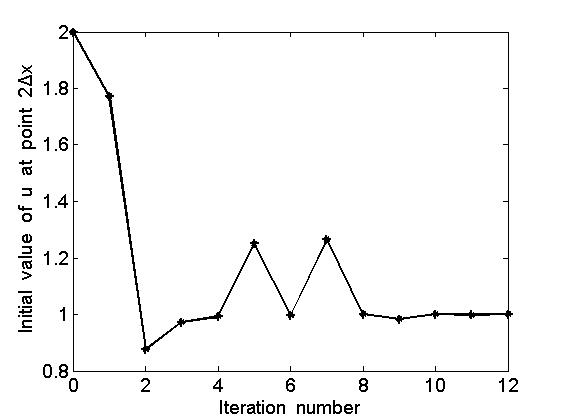

Supplement: S7 File — This file Includes all the result and figures used in the manuscript. (ZIP) [file pone.0191714.s007.zip › minor revision/figures/parabolic/2.jpg]

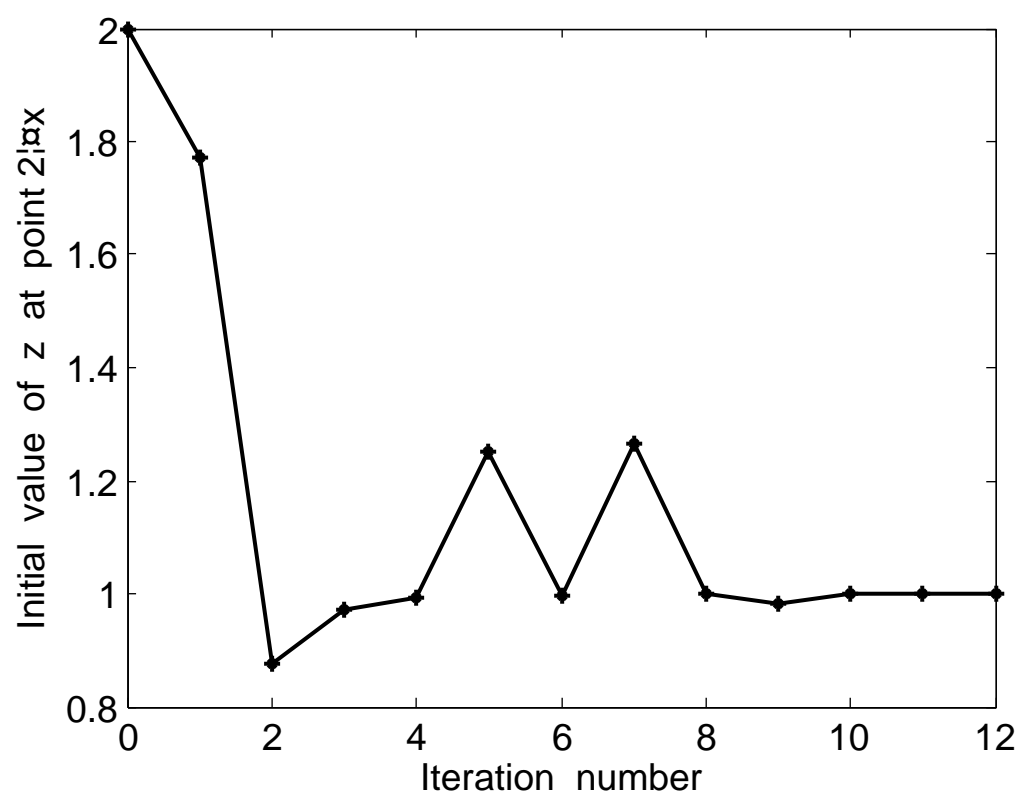

Supplement: S7 File — This file Includes all the result and figures used in the manuscript. (ZIP) [file pone.0191714.s007.zip › minor revision/figures/parabolic/2.pdf]

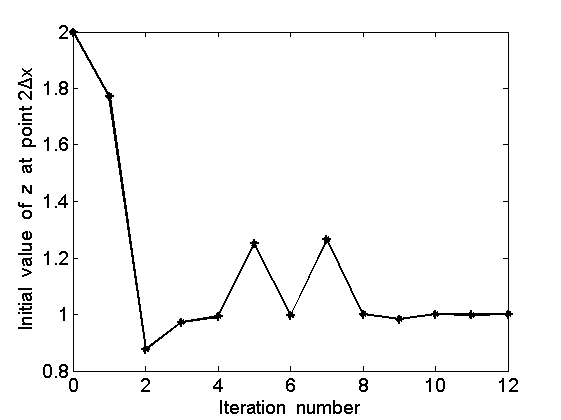

Supplement: S7 File — This file Includes all the result and figures used in the manuscript. (ZIP) [file pone.0191714.s007.zip › minor revision/figures/parabolic/2.png]

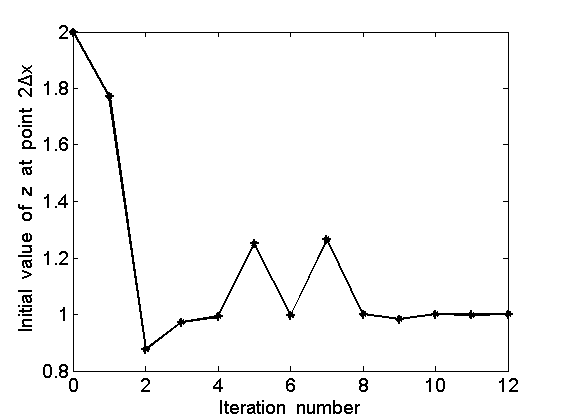

Supplement: S7 File — This file Includes all the result and figures used in the manuscript. (ZIP) [file pone.0191714.s007.zip › minor revision/figures/parabolic/2.tif]

Initial value of  $u$  at point  $2\Delta x$

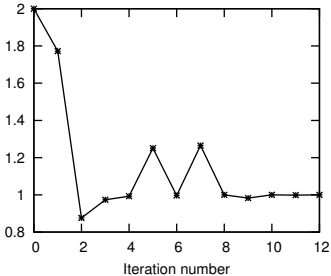

Supplement: S7 File — This file Includes all the result and figures used in the manuscript. (ZIP) [file pone.0191714.s007.zip › minor revision/figures/parabolic/22-eps-converted-to.pdf]

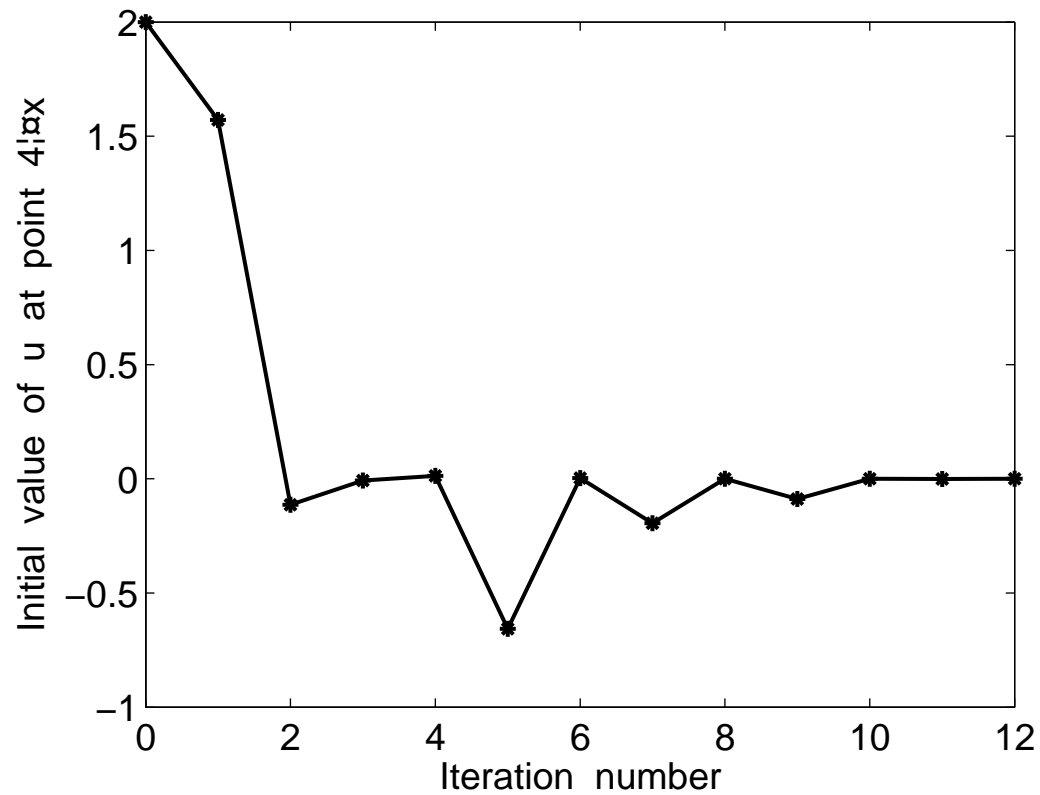

Supplement: S7 File — This file Includes all the result and figures used in the manuscript. (ZIP) [file pone.0191714.s007.zip › minor revision/figures/parabolic/4-eps-converted-to.pdf]

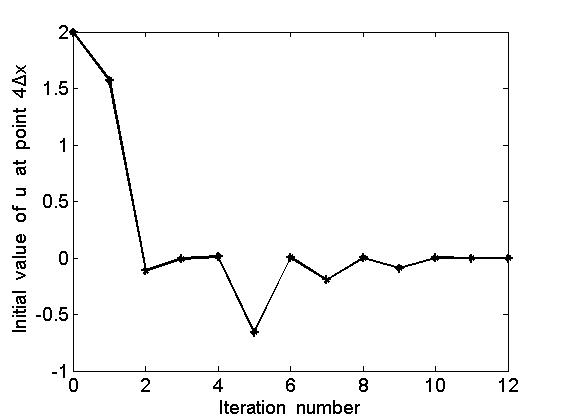

Supplement: S7 File — This file Includes all the result and figures used in the manuscript. (ZIP) [file pone.0191714.s007.zip › minor revision/figures/parabolic/4.jpg]

Initial value of  $u$  at point  $4\Delta x$

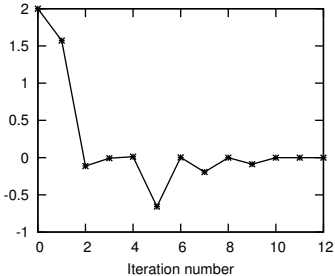

Supplement: S7 File — This file Includes all the result and figures used in the manuscript. (ZIP) [file pone.0191714.s007.zip › minor revision/figures/parabolic/44-eps-converted-to.pdf]

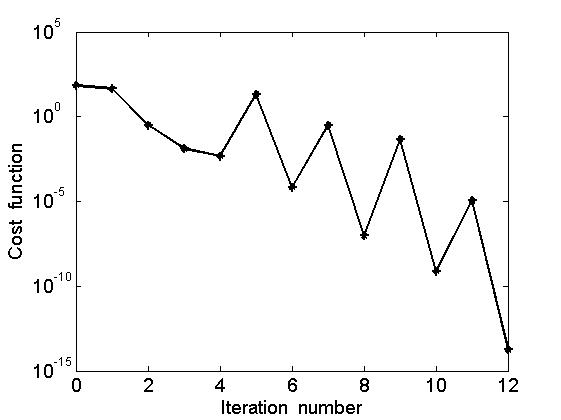

Supplement: S7 File — This file Includes all the result and figures used in the manuscript. (ZIP) [file pone.0191714.s007.zip › minor revision/figures/parabolic/cost.jpg]

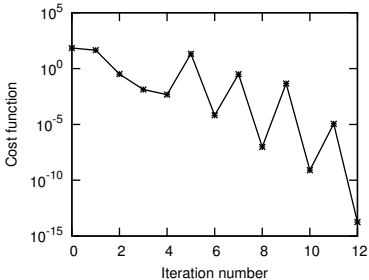

Supplement: S7 File — This file Includes all the result and figures used in the manuscript. (ZIP) [file pone.0191714.s007.zip › minor revision/figures/parabolic/Costfunction-eps-converted-to.pdf]

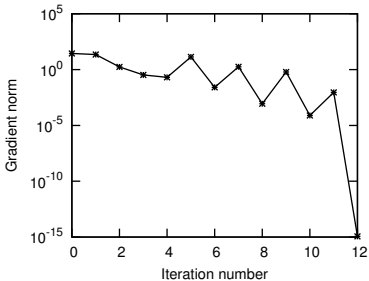

Supplement: S7 File — This file Includes all the result and figures used in the manuscript. (ZIP) [file pone.0191714.s007.zip › minor revision/figures/parabolic/Gradientnorm-eps-converted-to.pdf]

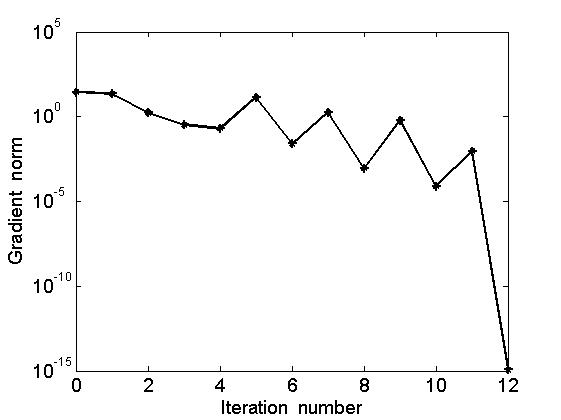

Supplement: S7 File — This file Includes all the result and figures used in the manuscript. (ZIP) [file pone.0191714.s007.zip › minor revision/figures/parabolic/norm.jpg]

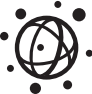

**PLOS**

**SUBMISSION**

Supplement: S7 File — This file Includes all the result and figures used in the manuscript. (ZIP) [file pone.0191714.s007.zip › minor revision/PLOS-submission-eps-converted-to.pdf]
